# Evolutionary multi-agent reinforcement learning for crisis-aware demographic policy optimization

**DOI:** 10.3389/fdata.2026.1842233

**Published:** 2026-07-08

**Authors:** Anton V. Dozhdikov, Arseniy M. Sitkovskiy

**Affiliations:** 1 Federal Research Sociological Centre of the Russian Academy of Sciences, Moscow, Russia; 2 Peoples Friendship University of Russia (RUDN University), Moscow, Russia

**Keywords:** computational demography, crisis modeling, crisis-aware simulation, demographic policy, evolutionary reinforcement learning, multi-agent reinforcement learning, reinforcement learning, Russia

## Abstract

Demographic systems face unprecedented challenges from simultaneous crises. Conventional statistical demography techniques and agent–based models often struggle to capture nonlinear inter–regional interactions during periods of severe socio–economic disruption. To address this, we propose *MADDPG–EVO–DGM*, a hybrid algorithm that integrates multi–agent deep reinforcement learning with evolutionary optimisation and meta–learning principles to model regional demographic processes under multiple crisis scenarios. Each region is treated as an autonomous agent learning to steer demographic policy levers, while periodic evolutionary “boosters” overcome local optima via population–based perturbations of actor network parameters. Additionally, a Darwin–Gödel Machine–inspired meta–learning mechanism adapts the booster triggers, enabling self–improvement in the learning process. We evaluate MADDPG–EVO–DGM on a simulation environment calibrated with real demographic data for eight federal regions of the Russian Federation over the period 2000–2024 and subject to ten concurrent crisis scenarios (e.g., pandemic, geopolitical conflict, economic collapse). Experiments demonstrate significantly faster convergence and improved performance over a baseline MADDPG: the hybrid approach achieves a higher final average reward (252.57 vs. 243.07) and 3.4 × lower convergence variance (σ = 0.24 vs. 0.80), indicating more reliable training. It also exhibits qualitative performance jumps of +68% during evolutionary phases and maintains 35%–45% greater resilience under crisis shocks compared to the baseline. To our knowledge, this is the first application of multi–agent reinforcement learning to large–scale demographic modeling under crises, opening new possibilities for evidence–based, crisis–resilient population policy design. Code, data, and logs are provided to ensure reproducibility.

## 1 Introduction

Demographic systems face unprecedented challenges from simultaneous crises. Conventional statistical demography techniques, such as the Lee–Carter model (Lee and Carter, 1992), and agent–based simulations (Billari and Prskawetz, 2003; Silverman et al., 2013) often struggle to capture nonlinear inter–regional interactions during periods of severe socio–economic disruption. The Russian Federation, with its 89 heterogeneous federal subjects, provides a compelling case: recent crises such as the COVID–19 pandemic (2020–2022), geopolitical instability with sanctions, and economic downturns have tested the resilience of regional populations in ways that static models cannot anticipate. This highlights a critical need for adaptive, data–driven approaches to demographic modeling under uncertainty.

Reinforcement learning (RL) offers a principled framework for dynamic decision–making in complex environments (Sutton and Barto, 2018). Multi–agent reinforcement learning (MARL) extends RL to systems of interacting decision–makers and is thus well–suited for modeling federated regions influencing each other's demographic outcomes (Zhang et al., 2021). However, straightforward application of MARL to demographic policy learning is difficult: the environment is non–stationary (policies of one region affect others), rewards are delayed due to the slow dynamics of population change, and naive gradient–based algorithms can become stuck in suboptimal equilibria. The MADDPG algorithm (Multi–Agent Deep Deterministic Policy Gradient) introduced by Lowe et al. (2017) addresses some non-stationarity issues via centralized training with decentralized execution, yet gradient–based MARL can still converge to suboptimal policies—particularly under complex, multi–crisis dynamics—because it may get trapped in local optima.

In this work we introduce *MADDPG–EVO*, a hybrid MARL approach augmented with evolutionary search, and its extended variant *MADDPG–EVO–DGM* that incorporates meta–learning elements inspired by the Darwin–Gödel Machine concept (Zhang et al., 2025). Evolutionary algorithms provide a global, population–based exploration that complements gradient descent by escaping local optima and injecting diversity into policies. Periodic evolutionary boosters are integrated into MADDPG training: after a fixed number of episodes, the current actor weights are mutated to create a population of candidate policies; the candidates are evaluated and the best performer replaces the incumbent policy. This intermittent evolutionary injection draws inspiration from prior hybrid algorithms (Khadka and Tumer, 2018; Pourchot and Sigaud, 2019; Majumdar et al., 2020) but is applied here to demographic modeling for the first time. Moreover, our MADDPG–EVO–DGM variant adapts this process with a meta–learning strategy: instead of a fixed booster schedule, the system monitors learning progress and triggers evolutionary search whenever improvement stagnates, echoing the self–modifying loop of a Darwin–Gödel Machine (Schmidhuber, 2007; Zhang et al., 2025). This represents an initial integration of Darwin–Gödel Machine principles—open-ended evolution and self-improvement—into a practical MARL algorithm for socio–demographic systems.

**Contributions**. Our contributions are fivefold:

1. **Crisis–aware demographic MARL**. We present the first application of MARL (MADDPG) to regional demographic and migration modeling at a country–wide scale, showing that agents can learn adaptive policies under multiple concurrent crisis scenarios.
2. **Evolutionary booster mechanism**. We integrate an evolutionary optimisation module into the MARL training loop to overcome local optima. The resulting MADDPG–EVO yields significant performance improvements and stability gains over standard MADDPG.
3. **Darwin–Gödel Machine augmentation**. We incorporate meta–learning principles inspired by the Darwin–Gödel Machine concept into the MARL framework—specifically allowing the algorithm to modify its own training dynamics (booster timing) based on performance—thereby enhancing exploration and convergence reliability.
4. **Crisis environment with real data**. We construct a novel environment with ten distinct crisis scenarios (pandemic, war, economic collapse, climate disaster, energy crisis, etc.) parameterised by their impacts on demographic rates, integrated with real historical data for multiple regions. This enables quantitative evaluation of crisis modifiers on population dynamics across eight representative regions over 25 years.
5. **Empirical evaluation**. Through extensive experiments we demonstrate that our hybrid methods improve outcomes: early–training rewards more than double with evolutionary boosters, the final average reward increases by approximately 4%, and there is a 3.4 × reduction in performance variance. We analyse training phases (pre–and post–booster) and show reliable convergence and heterogeneous agent behaviors, highlighting policy implications for regional demographic management. We further compare against a pure evolutionary baseline and an independent learning baseline (see Appendix B.4) and confirm that both lag far behind our hybrid methods, underscoring the necessity of combining gradient learning with evolution.

Although we restrict our experiments to eight representative regions for computational feasibility, these regions were chosen to reflect a diverse cross–section of socio-economic profiles (urban, rural, and industrial). The underlying algorithm and environment scale naturally to a larger number of agents via parameter sharing and parallel computation, albeit at increased computational cost.

## 2 Related work

### 2.1 Demographic modeling under crises

Traditional demographic forecasting methods [e.g., the Lee–Carter model (Lee and Carter, 1992)] rely on statistical extrapolation and often struggle with structural breaks during crises. Agent–based models have been employed to capture interactions and emergent phenomena in demographic systems (Billari and Prskawetz, 2003; Silverman et al., 2013), but such methods typically do not incorporate decision–making or multi–agent interactions. Recent studies have begun exploring machine–learning approaches for demographic modeling: Bohk-Ewald et al. (2018) applied predictive modeling to mortality and fertility rates, but such methods typically do not incorporate policy optimisation. Our work differs by focusing on policy optimisation in a multi–region system using MARL to actively learn how regional governments could respond to crisis shocks rather than merely forecasting population metrics.

### 2.2 Multi–agent reinforcement learning

MARL has advanced rapidly in domains such as games, robotics and traffic control, but its use in social systems modeling remains nascent. Algorithms like MADDPG (Lowe et al., 2017), COMA (Foerster et al., 2018), and QMIX (Rashid et al., 2018) address challenges such as non-stationarity and credit assignment. MADDPG in particular enables continuous action coordination via centralized critics and has shown effectiveness in mixed cooperative–competitive tasks. To our knowledge MARL has not previously been applied to computational demography. In our formulation, each region is a learning agent—conceptually related to agent–based models but with agents learning optimal strategies rather than following fixed rules. MARL allows agents to implicitly learn migration dynamics by optimizing their own region's outcomes in the context of other agents' actions. Early work applying MARL to socio–economic settings (e.g., coordinating tax policies or economic games) is beginning to emerge, but these efforts are still limited in scope. Our work is among the first to bring MARL to the domain of demography.

### 2.3 Hybrid evolutionary reinforcement learning

There is growing evidence that combining evolutionary algorithms with RL can yield improved exploration and performance on challenging tasks. Khadka and Tumer (2018) and Majumdar et al. (2020) showed that evolutionary population search can assist multi–agent coordination by providing a diverse set of experiences. Similarly, Pourchot and Sigaud (2019) introduced a genetic algorithm alongside policy gradients to escape local optima in continuous control tasks. These hybrid approaches exploit the complementary strengths of evolution (global search, diversity) and gradient descent (efficient fine–tuning). We integrate an intermittent evolutionary booster into MADDPG training for the first time in a demographic context. Unlike methods that run evolution continuously or in parallel, our booster monitors learning progress and triggers an evolutionary search whenever improvement stalls, injecting diversity into a slowly evolving environment. Furthermore, inspired by open–ended learning frameworks, we allow the booster schedule to adapt based on learning progress in our DGM–augmented variant. This aligns with the concept of a self–improving Darwin–Gödel Machine recently proposed by Zhang et al. (2025), which advocates an agent architecture capable of modifying its own algorithms through an evolutionary search process. Our approach takes a step in this direction by enabling the training algorithm itself to evolve (in terms of when and how it explores), not just the policy parameters.

### 2.4 Comparison with population–based training

While several *population–based training* (PBT) and auto–RL frameworks also combine evolutionary search with gradient learning, they operate quite differently from our method. PBT and related approaches such as CEM–RL or evolution strategies maintain multiple independent learner populations and periodically copy weights or hyperparameters between them on a fixed schedule to explore hyperparameter spaces (Jaderberg et al., 2017; Salimans et al., 2017). In contrast, our booster perturbs a *single* shared policy network across all agents and is *triggered adaptively* whenever the moving average of the reward stops improving. This design yields a more sample–efficient adaptation suited to cooperative multi–agent settings. To our knowledge evolutionary boosters have not previously been applied to MARL, particularly in the domain of demographic policy modeling.

## 3 Datasets, crisis scenarios, and regional sample

The empirical input file regions_data_selective.csv contains aggregated regional demographic and socio-economic indicators for Russian federal subjects; the crisis file crisis.txt contains the scenario definitions used by the simulator. The data source and variable structure are consistent with official aggregate regional statistical indicators published through Rosstat and the Unified Interdepartmental Statistical Information System (EMISS), including the regional socio-economic indicator series used for population, labor-market and economic variables (Federal State Statistics Service (Rosstat), 2026b,a). The modeling dataset contains no individual-level records (Table 1).

**Table 1:** Columns in the regional input dataset.

Column	Type	Role in modeling
Region_name	Categorical	Name of the federal subject/regional agent.
Year	Integer	Calendar year of the regional observation.
Region_id	Integer	Numeric regional identifier used for indexing.
Population	Numeric	Regional population stock and population-stability calculations.
Birth_rate	Numeric	Fertility/vital-rate state component affected by family-support actions and crisis modifiers.
Death_rate	Numeric	Life expectancy at birth, used as an inverse summary indicator of mortality; higher values indicate lower mortality.
Natural_increase_rate	Numeric	Derived demographic-balance indicator used in the state and reward.
Migration_balance	Numeric	Net migration component used in the state and reward.
Gdp_per_capita	Numeric	Economic state component affected by the economic-support policy lever and crisis modifiers.
Unemployment_rate	Numeric	Labor-market state component used in the reward.
Average_wage	Numeric	Income proxy used as an economic state component.

Monetary variables retain the units of the source file. The column death_rate contains life expectancy at birth and is used as an inverse mortality indicator.

**Figure 1:**
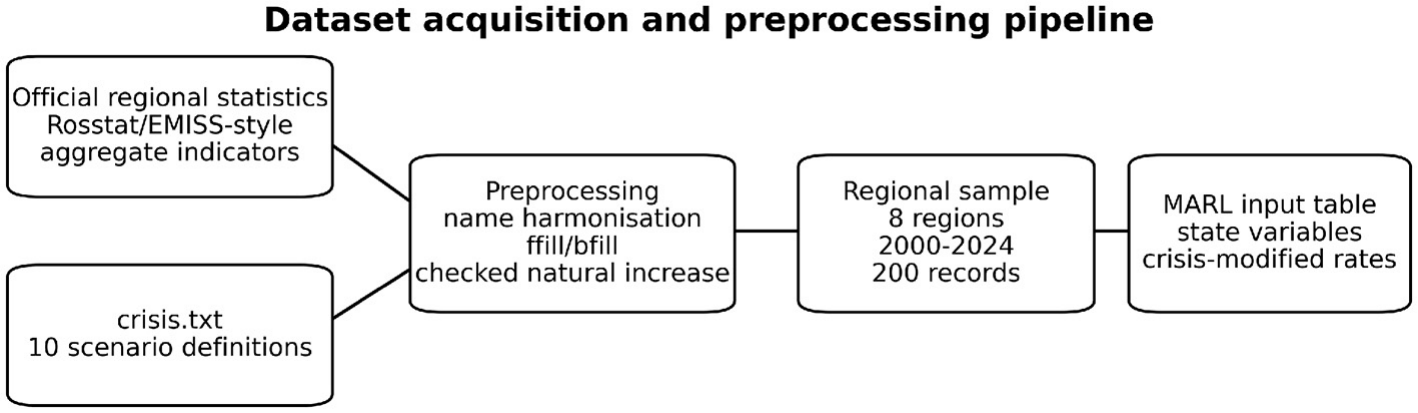
Dataset acquisition and preprocessing pipeline. Regional statistics and the external crisis-scenario file are converted into the tabular input used by the MARL environment.

The notebook preprocesses the regional file by forward- and backward-filling missing aggregate values, checking natural-increase values where necessary, and constructing regional statistical profiles. In the input file, the column labeled death_rate contains life expectancy at birth; it is used as an inverse summary indicator of mortality conditions rather than as a crude death rate. Higher life expectancy therefore represents lower mortality in the demographic interpretation. The modeling subset used in the main experiments consists of eight regions and annual observations for 2000–2024, giving 200 region-year records before scenario expansion. Table 2 gives a 1-year snapshot of the selected regional records, and Table 3 reports descriptive statistics over the selected 2000–2024 input subset before crisis modifiers are applied (Figure1).

**Table 2:** Snapshot of the selected regional dataset for year 2000, before crisis modifiers.

Region	Pop.	Birth	Life exp.	Migration	GDP pc	Unemp.	Wage
Moscow	9.93	0.980	69.8	66,665	115.6	2.5	18.4
St. Petersburg	4.74	0.933	66.7	11,422	39.8	3.0	42.8
Tatarstan	3.79	1.292	67.6	8,724	49.1	3.1	43.6
Krasnodar Krai	5.13	1.257	67.1	22,560	26.7	3.1	34.5
Sverdlovsk Oblast	4.58	1.126	63.7	7,234	34.2	3.8	41.6
Novosibirsk Oblast	2.73	1.125	66.3	5,508	26.5	2.3	41.2
Samara Oblast	3.29	1.056	64.5	12,786	42.8	2.8	32.9
Rostov Oblast	4.45	1.116	66.5	7,669	20.0	2.9	27.5

The life-expectancy column corresponds to the source-file field death_rate. Population and GDP per capita are divided by 10^6^ and 10^3^, respectively, for compact display.

**Table 3:** Descriptive statistics for the selected eight-region input subset, 2000–2024, before crisis modifiers.

Variable	Mean	SD	Median	Min.	Max.
Population, million	5.08	2.68	4.31	2.64	13.15
Birth rate	1.426	0.212	1.412	0.933	1.942
Life expectancy	70.72	3.37	70.80	63.70	79.38
Natural increase	-0.98	4.95	-1.93	–14.26	26.59
Migration balance, thousand	19.25	24.82	8.95	–5.13	112.21
GDP per capita, thousand	397.62	344.76	342.63	20.00	1,567.65
Unemployment rate	3.12	0.96	3.00	1.30	6.70
Average wage	45.19	13.11	43.65	9.10	69.00

Life expectancy is used as an inverse summary indicator of mortality conditions. Population and GDP per capita are displayed in millions and thousands, respectively; migration balance is displayed in thousands.

**Figure 2:**
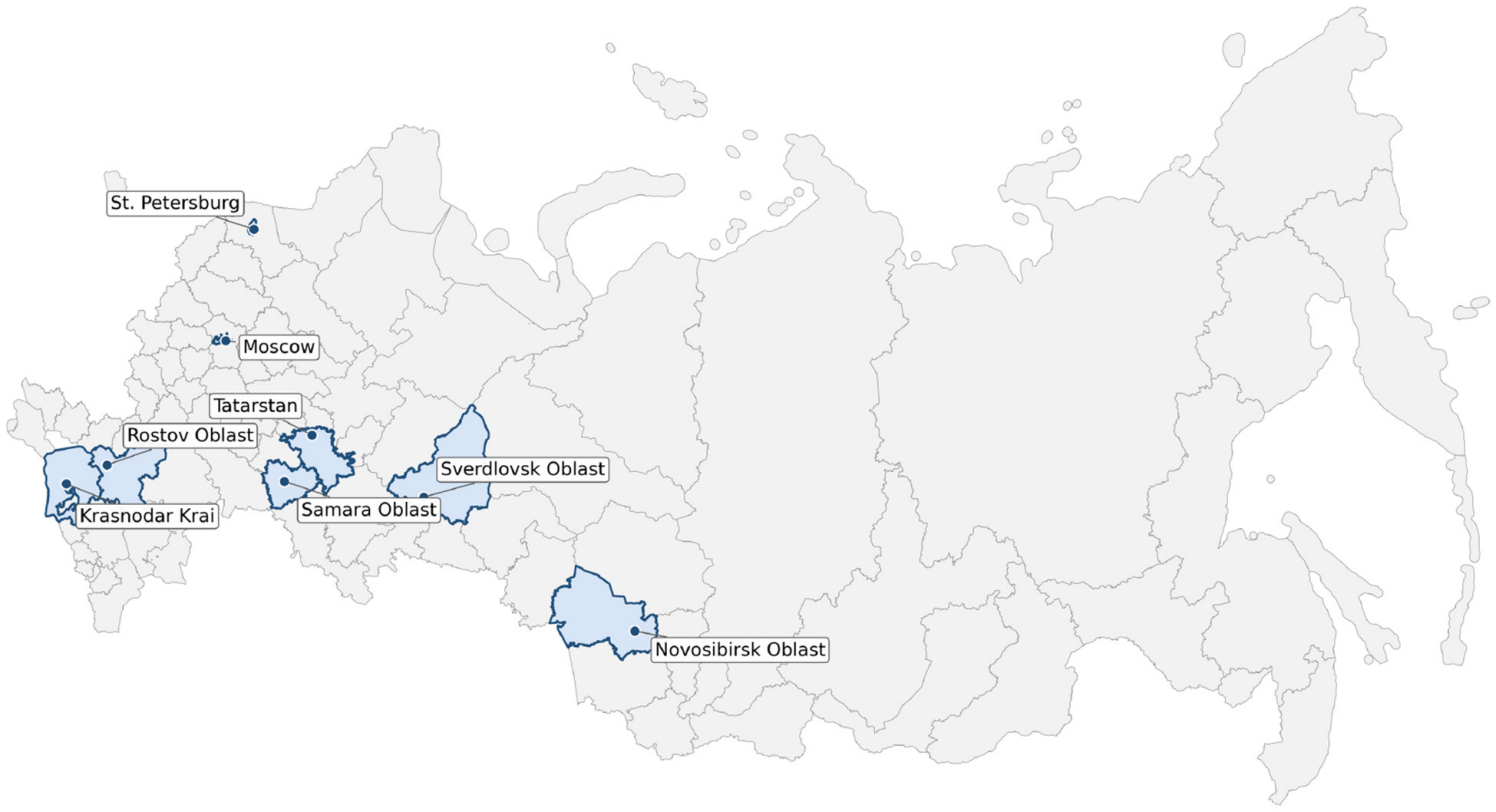
Single-panel labeled map of the eight representative regional agents. Boundaries are drawn from the supplied regional GeoJSON file included in the reproducibility package; selected federal subjects are highlighted, and markers show administrative-center coordinates for visual placement at national scale.

The eight-region sample was chosen as a purposive computational sample rather than as a statistically representative sample of all federal subjects. It combines two largest metropolitan poles (Moscow and St. Petersburg), a large Volga republic and industrial center (Tatarstan), a southern migration-attractive and agrarian-industrial region (Krasnodar Krai), an Urals industrial region (Sverdlovsk Oblast), a Siberian metropolitan/industrial region (Novosibirsk Oblast), and two large Volga/Southern industrial-agricultural regions (Samara and Rostov oblasts). This combination was selected to expose the MARL system to heterogeneous population size, migration, labor-market and income profiles while keeping the training problem computationally feasible for eight simultaneous agents (Figure 2).

**Figure 3:**
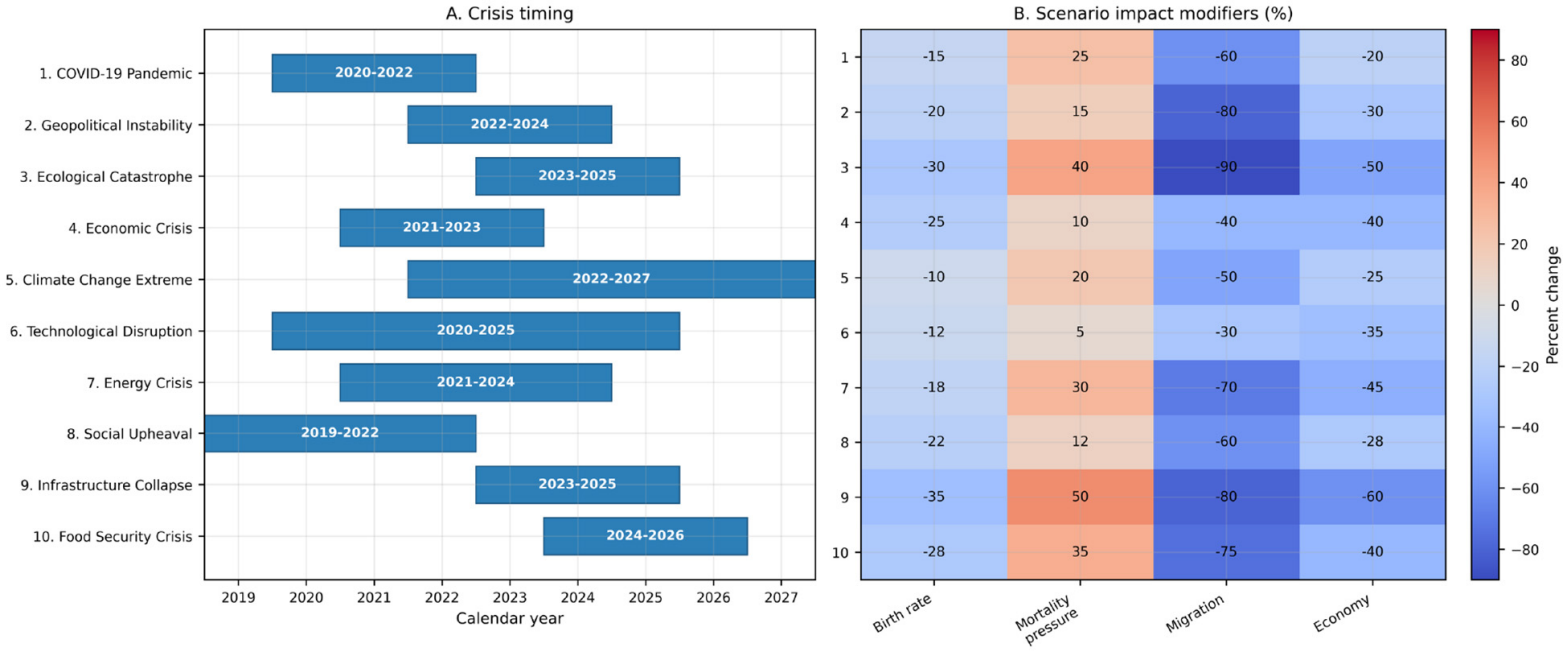
Diagrammatic representation of the ten crisis scenarios. **(A)** Shows the timing and overlap of the scenarios; **(B)** shows the four full-intensity impact modifiers in the scenario file, with mortality pressure interpreted through the life-expectancy proxy.

The crisis-scenario file contains ten stylised exogenous shocks. Each scenario has a start year, an end year, a short description and four impact modifiers: birth-rate change, mortality-pressure change, migration change and economic impact. The scenario-file variable death_rate_change denotes adverse or favorable pressure on mortality conditions and is interpreted through the life-expectancy-based mortality indicator described above. A positive mortality-pressure coefficient corresponds to deteriorating mortality conditions, i.e., lower life expectancy in the inverse-indicator interpretation. When several scenarios overlap in a given year, their modifiers are applied sequentially by the preprocessing code, so the effective shock can be stronger than any single scenario alone. The scenarios are intentionally stylised stress tests rather than forecasts of realized events (Figure 3).

## 4 Methodology

**Figure 4:**
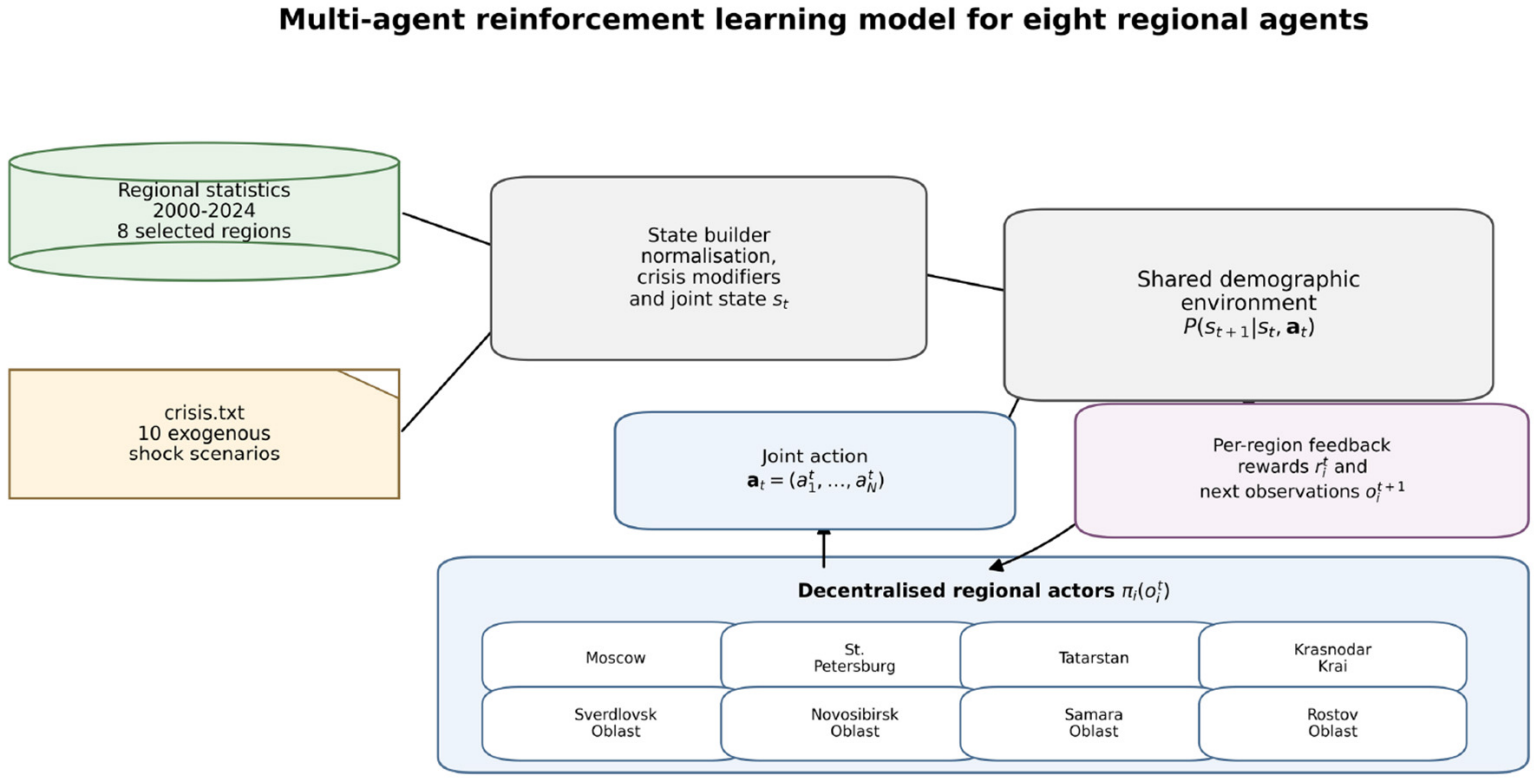
Multi-agent reinforcement-learning model used in the demographic experiments. Eight regional agents act simultaneously in a shared demographic environment; the regional input table and external crisis-scenario file define the initial state and shock structure.

We model demographic dynamics across multiple regions as a multi–agent sequential decision process. The environment describes the crisis environment and data integration, the state and action spaces, the MADDPG architecture and training procedure, and the evolutionary booster mechanism. Figure 4 gives an overview of the multi-agent model before the individual MDP components are detailed below.

### 4.1 Crisis–aware demographic environment

**Figure 5:**
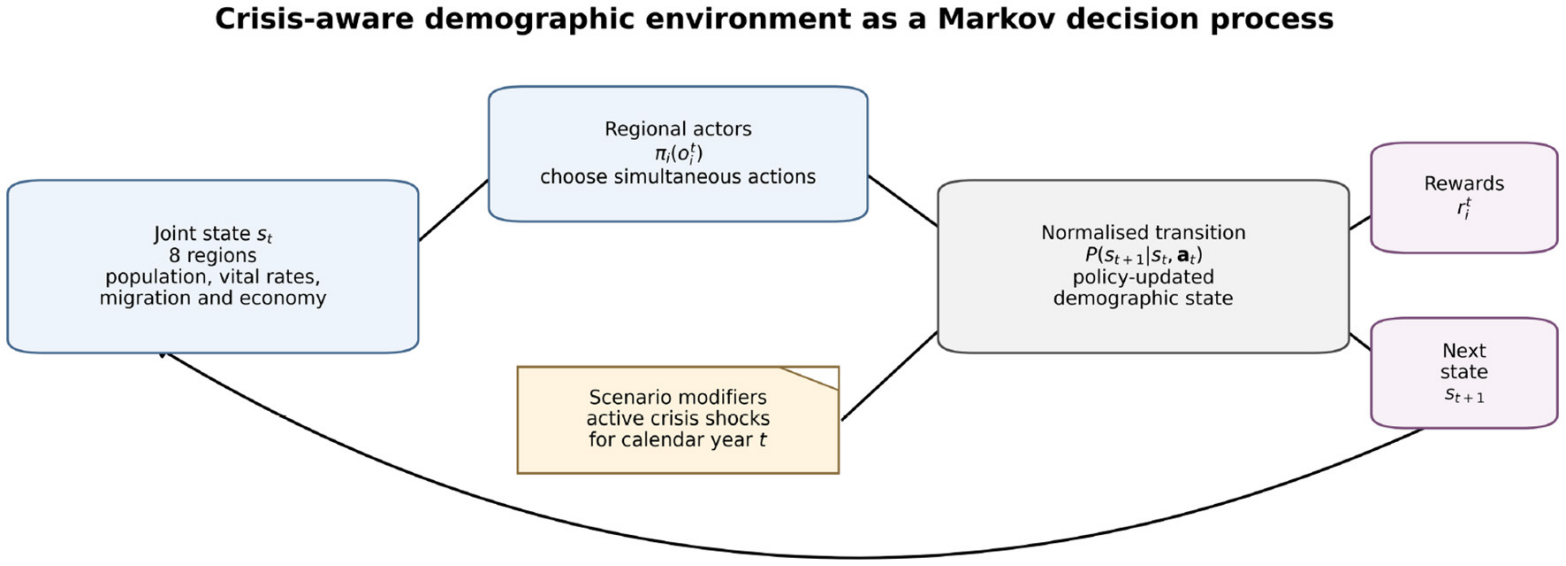
Crisis-aware demographic environment represented as an MDP. The input-generation layer applies exogenous scenario modifiers to regional demographic and economic variables; the RL step then maps the current joint state and simultaneous four-dimensional regional actions into next states and per-region rewards.

Our environment simulates population and migration dynamics across *N* regions on annual time steps. Baseline demographic and socio-economic inputs, the eight-region sample and all ten crisis scenarios are documented in Section 3. In the main experiments we use *N* = 8 regional agents for computational feasibility. The preprocessing pipeline constructs the modeling input for 2000–2024 and applies the crisis modifiers during input generation whenever a scenario is active in a calendar year. The MARL environment then evolves the resulting normalized state vectors through the learned policy-transition system; scenario labels are not supplied to agents as separate observed variables. The crisis scenarios should therefore be interpreted as stress-test modifiers of the empirical state distribution rather than as forecasts of named events (Table 4). Figure 5 gives the corresponding MDP view of the environment.

**Table 4:** Ten crisis scenarios used in the scenario file.

**No**.	Scenario	Years	Description	Modifiers
1	COVID-19 pandemic	2020–2022	Lockdowns, increased mortality, and reduced mobility.	B −15%; D +25%; M −60%; E −20%.
2	Geopolitical instability	2022–2024	Conflict, sanctions, mobilization, and brain drain.	B −20%; D +15%; M −80%; E −30%.
3	Ecological catastrophe	2023–2025	Major environmental disaster such as nuclear accident, oil spill, or wildfire.	B −30%; D +40%; M −90%; E −50%.
4	Economic crisis	2021–2023	Severe recession, hyperinflation, and unemployment.	B −25%; D +10%; M −40%; E −40%.
5	Climate change extreme	2022–2027	Extreme weather events, droughts, floods, and permafrost melting.	B −10%; D +20%; M −50%; E −25%.
6	Technological disruption	2020–2025	Automation, AI-related job replacement, and social unrest.	B −12%; D +5%; M −30%; E −35%.
7	Energy crisis	2021–2024	Energy shortages, infrastructure stress, and heating crisis.	B −18%; D +30%; M −70%; E −45%.
8	Social upheaval	2019–2022	Political instability, protests, and institutional breakdown.	B −22%; D +12%; M −60%; E −28%.
9	Infrastructure collapse	2023–2025	Failure of transport, healthcare, or utility infrastructure.	B −35%; D +50%; M −80%; E −60%.
10	Food security crisis	2024–2026	Agricultural failure, shortages and supply-chain disruption.	B −28%; D +35%; M −75%; E −40%.

Full-intensity modifiers are percentages for B, birth rate; D, mortality pressure interpreted through the life-expectancy proxy; M, migration balance; E, economic component.

The environment state $s_{i}^{t}$ for region *i* at time *t* includes current population and vital-rate indicators as well as economic indicators: birth rate, life expectancy as an inverse mortality indicator, natural increase, net migration, GDP per capita, unemployment, population and average wage. The demographic interpretation follows the balance identity in Equation (1)


$$\begin{array}{l} {\text{population}}_{i}\left(t+1\right)={\text{population}}_{i}\left(t\right)+{\text{births}}_{i}^{t}-{\text{deaths}}_{i}^{t}+{\text{migrants}}_{i}^{t}, \end{array}$$ (1)

while the simulation implementation works on normalized state components. Specifically, the action vector modifies the normalized birth-rate, life-expectancy/mortality, migration and economic components; the natural-increase component is updated together with the demographic state; and the normalized population component is updated from the resulting natural-increase and migration components. Inter-regional context enters the MARL problem through each agent's observation, which contains both its own state and the average state of the other regions, and through centralized critics during training. The current implementation does not impose an explicit origin–destination migration-flow conservation constraint; adding such a flow model is an important extension for future work. Each simulation episode uses a fixed 50-step rollout over the normalized annual-transition dynamics, allowing long-term evaluation of policy effects from the initial regional state.

### 4.2 Agents: actions, observations, and rewards

Each region's government is modeled as an agent with a continuous four-dimensional action vector in Equation (2)


$$\begin{array}{l} {\text{a}}_{i}^{t}=\left(a_{i,F}^{t},a_{i,H}^{t},a_{i,M}^{t},a_{i,E}^{t}\right)\in {\left[-1,+1\right]}^{4}, \end{array}$$ (2)

where the four aggregate policy levers represent family/fertility support, healthcare and life-expectancy improvement (mortality reduction), migration attractiveness, and economic/labor-market support. Positive values intensify the corresponding lever, while negative values reduce or restrict it. This vector representation is important: the government policy in the model is not a single number. For example, an agent can simultaneously increase healthcare support $\left(a_{i,H}^{t}>0\right)$ and restrict migration attractiveness $\left(a_{i,M}^{t}<0\right)$. The action space remains intentionally aggregated, but it is sufficient to represent simultaneous policy directions across the four dimensions used in the implementation. All agents act simultaneously at each time step, and the environment updates the state of all regions for the next year accordingly.

#### 4.2.1 Demographic policy modeling

In this article, “policy” means a low-dimensional control vector in a simulation model, not a complete legal or administrative programme. Each action component changes a normalized demographic or economic state component: the fertility lever changes the birth-rate component, the healthcare lever improves the life-expectancy-based mortality component, the migration lever changes net migration, and the economic lever changes GDP per capita and unemployment. The resulting state transition is then evaluated through the reward function. This formulation is a standard MDP abstraction: it is designed to compare adaptive decision rules under common assumptions, not to prescribe concrete policy packages.

An agent's observation $o_{i}^{t}$ consists of its own normalized state $s_{i}^{t}$ and the average state of the other regions, producing a compact local-plus-system context. Agents do not observe other agents' actions directly; inter-regional context is available through the aggregate state component of the observation and through the centralized critic during training. The reward for each agent is designed to reflect desirable demographic and economic changes for its region. In the implementation, the per-region reward is computed on normalized state components as shown in Equations (3) and (4)


$$\begin{array}{l} r_{i}^{t}=10\left(b_{i}^{t+1}-b_{i}^{t}\right)+10\left(\ell_{i}^{t+1}-\ell_{i}^{t}\right)+15\left(n_{i}^{t+1}-n_{i}^{t}\right) \end{array}$$ (3)

$$\begin{array}{l} +5\left(m_{i}^{t+1}-m_{i}^{t}\right)+5\left(g_{i}^{t+1}-g_{i}^{t}\right)+5\left(u_{i}^{t}-u_{i}^{t+1}\right)\\ -5|p_{i}^{t+1}-p_{i}^{t}|, \end{array}$$ (4)

where *b*, ℓ, *n*, *m*, *g*, *u*, and *p* denote the normalized birth-rate, life-expectancy, natural-increase, migration, GDP-per-capita, unemployment and population components, respectively. Life expectancy is used here as an inverse mortality indicator: higher ℓ corresponds to lower mortality conditions. The signs encode the desired direction: higher birth rate, life expectancy, natural increase, migration and GDP increase reward, lower unemployment increases reward, and excessive population volatility is penalized.

As a numerical illustration, suppose that one transition increases the normalized birth-rate component by 0.010, increases the life-expectancy component by 0.005, increases natural increase by 0.015, increases migration by 0.020, increases GDP by 0.010, reduces unemployment by 0.003, and changes the population component by 0.004. The reward contribution is given in Equation (5).


$$\begin{array}{r} 10\left(0.010\right)+10\left(0.005\right)+15\left(0.015\right)+5\left(0.020\right)+5\left(0.010\right)\\ +5\left(0.003\right)-5\left(0.004\right)=0.520. \end{array}$$ (5)

**Figure 6:**
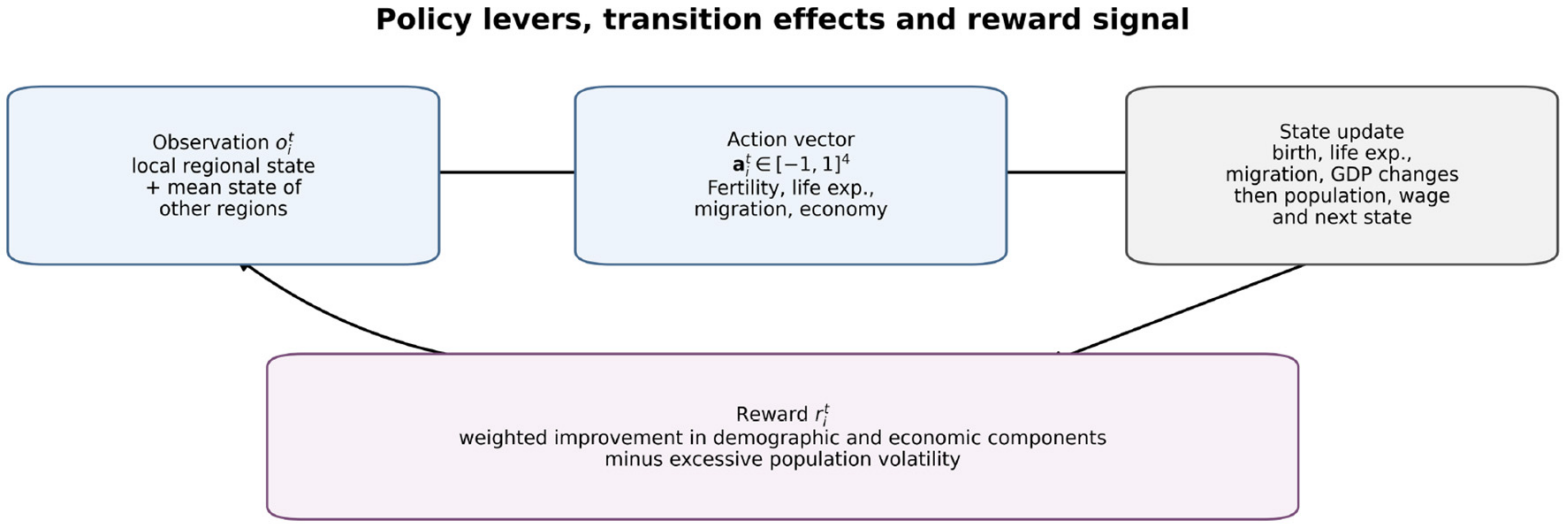
Diagrammatic representation of the action vector, state-transition effects and reward signal. This figure clarifies that the policy control is four-dimensional rather than a single scalar.

Figure 6 summarizes the action, transition and reward structure.

Each agent seeks to maximize its discounted cumulative reward in Equation (6).


$$\begin{array}{l} G_{i}^{t}=\overset{\infty }{\underset{k=0}{\sum }}\gamma^{k}r_{i}^{t+k}, \end{array}$$ (6)

with discount factor γ = 0.95. This discounted-return objective is how delayed rewards enter the model. If a healthcare action is reduced at time *t* but an economic or fiscal lever is increased after a lag of *k* steps, the later outcome contributes to the current policy evaluation through the term $\gamma^{k}r_{i}^{t+k}$. The present implementation does not introduce explicit policy-specific administrative lags beyond the state-transition sequence and discounting; adding separate lag parameters for particular policy levers is a natural extension.

While the reward drives learning, we also evaluate a *stability metric* defined as the inverse coefficient of variation of the total population. This demography-inspired measure quantifies how steady the population remains over time; it is used solely for *post-hoc* analysis of policy behavior and is *not* part of the agents' optimisation objective.

### 4.3 MADDPG architecture and training

**Figure 7:**
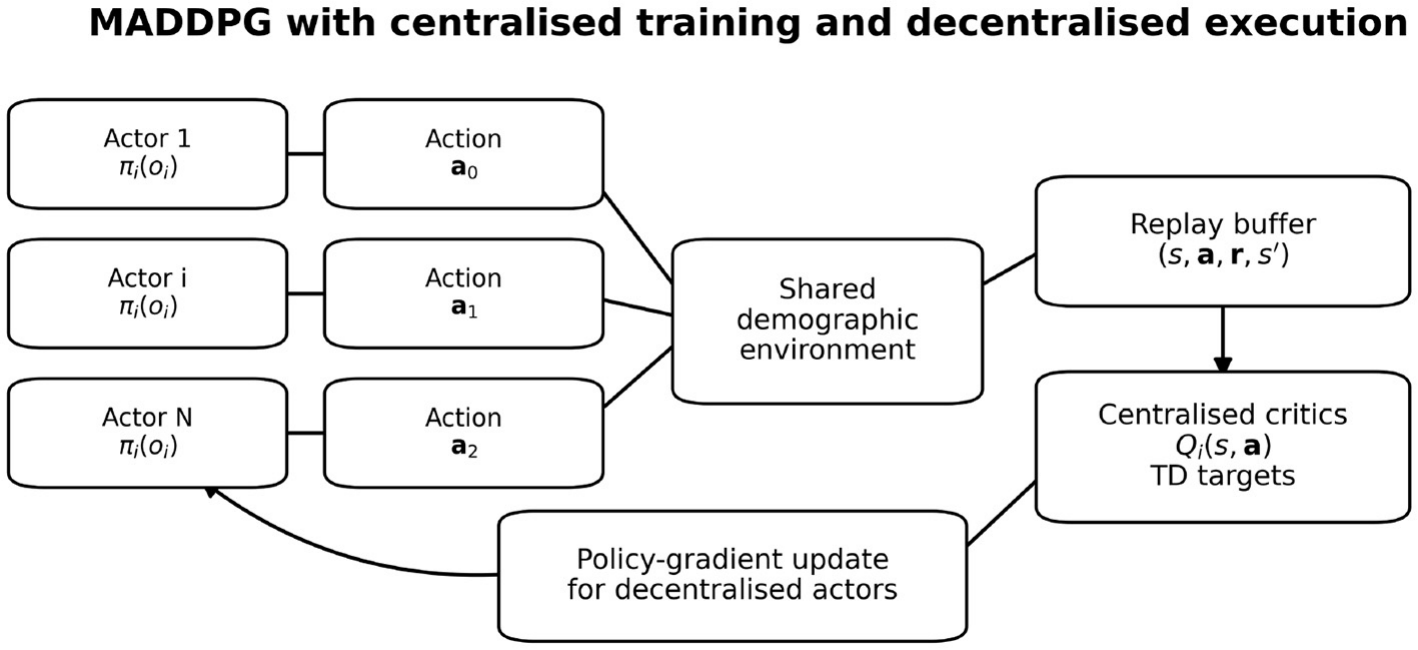
MADDPG architecture under centralized training and decentralized execution. Actors map regional observations to four-dimensional actions; critics are trained with joint state-action information through the replay buffer and TD targets.

We adopt the MADDPG algorithm (Lowe et al., 2017), an extension of deterministic policy gradient methods to multi–agent settings. Each agent *i* has an *actor* network π_ϕ_*i*__ that maps its observation $o_{i}^{t}$ to an action $a_{i}^{t}$, and a *critic* network *Q*_θ_*i*__ that estimates the Q–value for agent *i* given the joint state and joint actions of all agents. During training, critics are centralized: $Q_{\theta_{i}}\left(s_{t},a_{1}^{t},\ldots ,a_{N}^{t}\right)$ takes as input the full state (all regions' states) and all agents' actions, which mitigates non–stationarity by giving each critic complete information about the environment at that time. Actors, however, are decentralized and use only local observations at execution time [each $\pi_{\phi_{i}}\left(o_{i}^{t}\right)$ depends only on region *i*'s information] (Figure 7).

We train the networks off–policy using experiences sampled from a replay buffer. Each experience consists of $\left(s_{t},{\left\{a_{i}^{t}\right\}}_{i=1}^{N},{\left\{r_{i}^{t}\right\}}_{i=1}^{N},s_{t+1}\right)$. The critic for agent *i* is updated by minimizing the temporal-difference (TD) error in Equation (7)


$$\begin{array}{r} L\left(\theta_{i}\right)=\text{𝔼}([r_{i}^{t}+\gamma Q_{\theta_{i}}^{\prime }\left(s_{t+1},\pi_{\phi_{1}}^{\prime }\left(o_{1}^{t+1}\right),\ldots ,\pi_{\phi_{N}}^{\prime }\left(o_{N}^{t+1}\right)\right)\\ -Q_{\theta_{i}}\left(s_{t},a_{1}^{t},\ldots ,a_{N}^{t}\right){)}^{2}], \end{array}$$ (7)

where $Q_{\theta_{i}}^{\prime }$ and $\pi_{\phi_{i}}^{\prime }$ are target networks (softly updated clones of the main networks). The actor for agent *i* is updated via the deterministic policy gradient, which is given by Equation (8).


$$\nabla_{\phi_{i}}J(\phi_{i})={\text{𝔼}}_{s\sim D}[\nabla_{a_{i}}Q_{\theta_{i}}(s,a_{1},\ldots ,a_{N}){|}_{a_{i}=\pi_{\phi_{i}}(o_{i})}\;\nabla_{\phi_{i}}\pi_{\phi_{i}}(o_{i})].$$ (8)

In practice we *share* the actor and critic networks among homogeneous agents for computational efficiency (i.e., a single policy network is used for all regions, with agent–specific inputs or ID embeddings to allow differentiation). This parameter sharing assumes that regions have similar action–state structure, which is reasonable in our case (all are regional governments with the same action definition). Sharing significantly reduces the number of parameters and helps with scalability to larger numbers of agents, at the cost of limiting policy heterogeneity somewhat (though regions can still behave differently due to different inputs and experiences).

### 4.4 Evolutionary booster mechanism

Standard gradient–based MARL can stagnate in complex, delayed–reward environments such as ours. To address this, we introduce an *evolutionary booster* that perturbs the policy weights and selects improved variants when learning progress stalls. If there is no progress, we pause regular training and perform an evolutionary search on the actor network parameters:

1. **Population generation**. Create a population of *M* mutated copies of the current actor network weights by adding independent Gaussian noise (mean 0) with standard deviation σ to each parameter. These *M* variants, plus optionally the unmutated incumbent, form the candidate set.
2. **Evaluation**. Evaluate each mutated policy in the environment for a short rollout (we use one episode per variant for efficiency), using the same mutation across all agents (i.e., all agents use the same mutated weights in a given evaluation rollout, preserving symmetry among agents).
3. **Selection**. Identify the variant achieving the highest average reward (aggregated across agents and time) in its evaluation rollout. If this best variant outperforms the incumbent policy, replace the actors' weights with this variant. We also retain a small elite set of top–performing mutants for potential reseeding (in case a future evolutionary cycle needs additional diverse starting points).
4. **Resume learning**. Continue MADDPG training from the (possibly updated) policy. The critic networks are *not* mutated; they are kept from before the booster so that value estimates remain intact and can rapidly adapt to the new policy.

**Figure 8:**
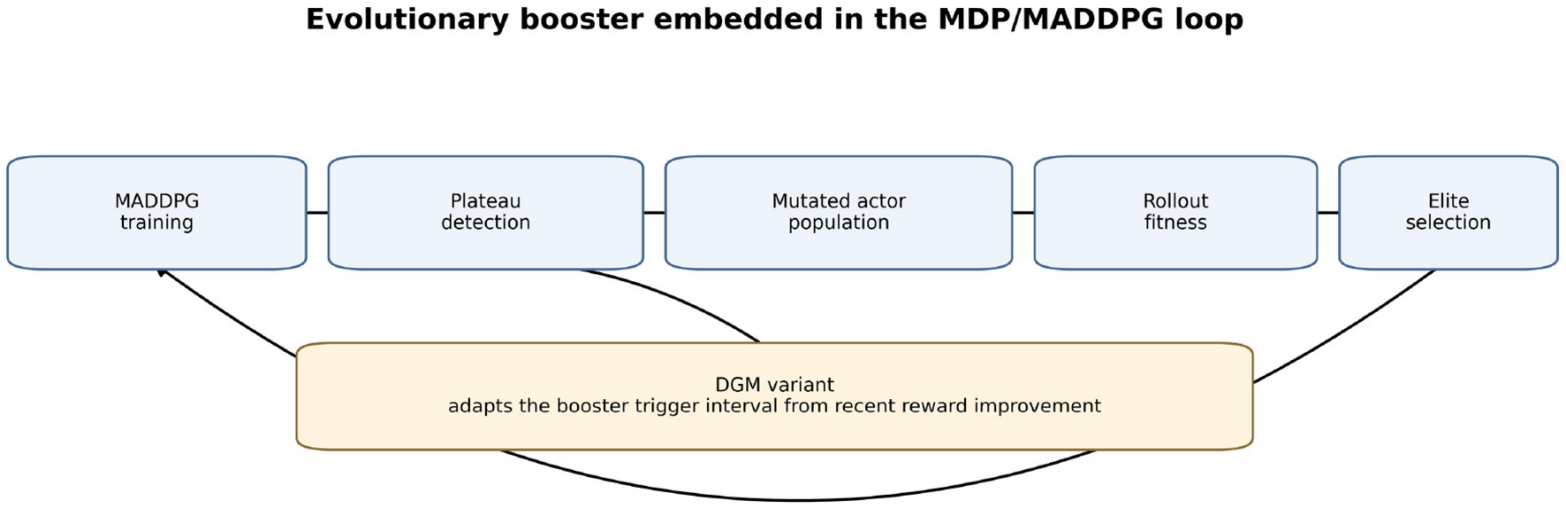
Evolutionary booster in the MDP/MADDPG loop. When progress stalls, actor parameters are mutated to form a candidate population; candidates are evaluated through rollouts, ranked by fitness, and the selected policy resumes gradient-based MADDPG training.

This intermittent evolutionary search injects diversity and allows the policy to jump out of local optima. Intuitively, gradient–based training gets the agents to a reasonably good set of policies, then the evolutionary booster shakes up the policy parameters in a coordinated way to explore very different behaviors—if any such behavior proves significantly better, the algorithm accepts it and then refines around that new policy with further gradient training (Figure 8).

#### 4.4.1 Meta–learning adaptation

**Figure 9:**
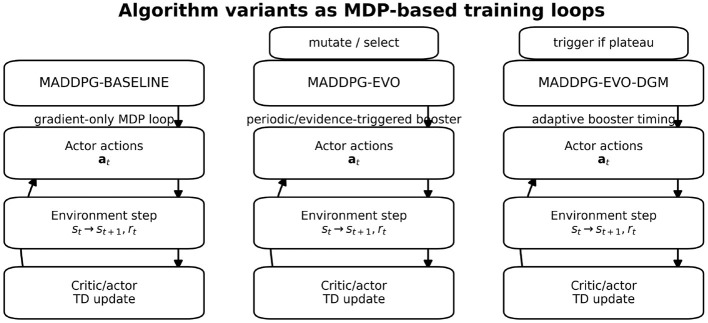
Diagrammatic comparison of MADDPG-BASELINE, MADDPG-EVO, and MADDPG-EVO-DGM as MDP-based training loops. The baseline uses only gradient-based actor/critic updates; EVO adds mutation-selection booster cycles; EVO-DGM adapts the booster timing based on reward stagnation.

In the MADDPG–EVO–DGM variant we introduce a simple meta–learning adaptation to the booster mechanism. Instead of using a fixed booster interval *K*, the algorithm monitors the recent improvement in average reward. If the rolling improvement over the last few episodes falls below a threshold (indicating learning has plateaued), the booster is triggered early; conversely, if learning is rapidly improving, the booster can be delayed to allow gradient ascent to continue. In our implementation we set a minimum interval of 20 episodes and then dynamically decide the next booster timing based on a moving average of reward gains. This adaptive schedule means the evolutionary intervention happens only when needed, akin to the Darwin–Gödel Machine idea of invoking self–modification when the current performance stalls. All other aspects of the booster (mutation generation, evaluation, and selection) remain the same. The DGM–inspired adaptation adds negligible overhead but yields a more flexible, self-tuning algorithm—an initial step toward open–ended self-improvement within our MARL training loop. We emphasize that our use of the Darwin–Gödel Machine notion is conceptual: the meta–learning adaptation adjusts booster timing based on observed performance rather than implementing the formal proof–search capabilities of a full Gödel machine. It should thus be viewed as a pragmatic, self-modifying heuristic inspired by the broader DGM philosophy (Figure 9).

## 5 Experiments and results

**Figure 10:**
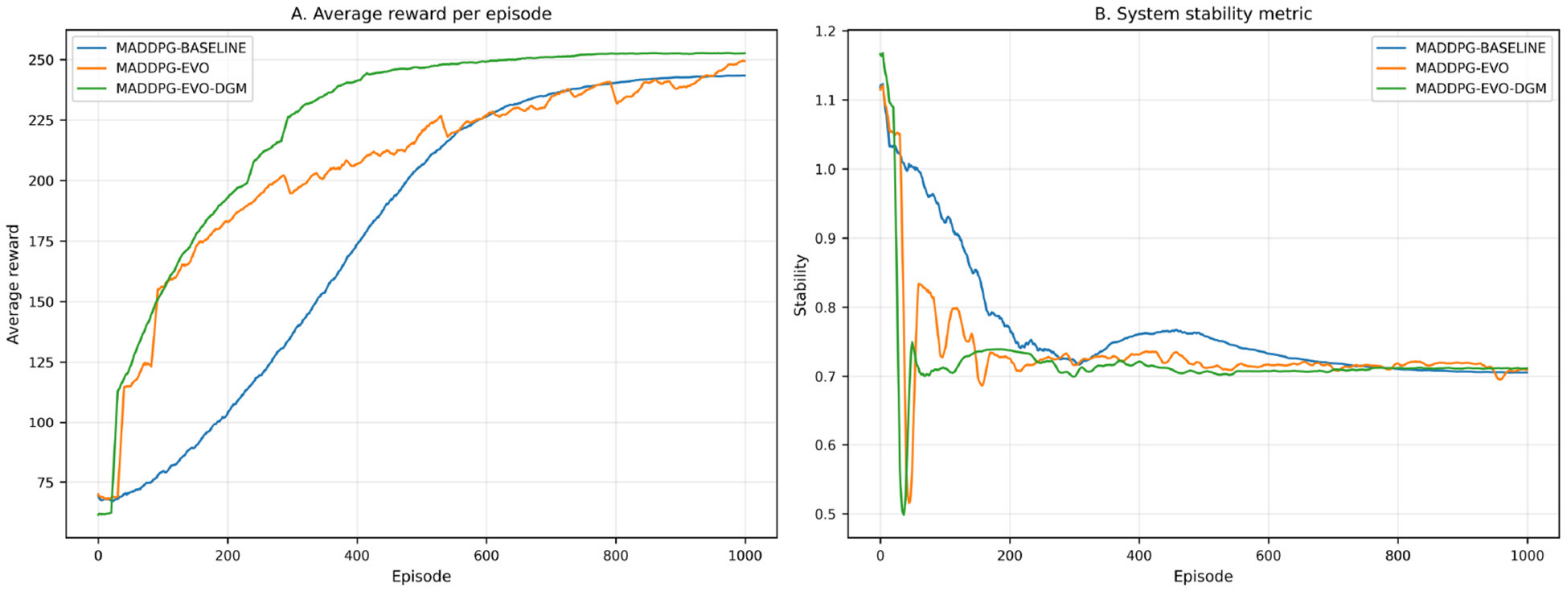
Training performance curves comparing the baseline MARL and the evolutionary approaches, derived from logged episode metrics. **(A)** Average reward per episode (higher is better). **(B)** System stability metric (inverse coefficient of variation of total population, higher indicates more stable population dynamics). Curves are shown as moving averages with a 10-episode window. The evolutionary methods achieve higher rewards faster and maintain stability, with the DGM variant reaching the highest asymptotic reward.

We conducted experiments comparing three approaches: (i) **MADDPG–BASELINE** (gradient–based MARL without evolutionary boosts), (ii) **MADDPG–EVO** (with evolutionary boosters triggered when learning progress stalls), and (iii) **MADDPG–EVO–DGM** (with an adaptive meta–learning booster schedule). We analyse learning dynamics, final performance and policy behaviors under various crises. Each training run involved eight regional agents (due to computational limits) controlling the selected regions as described in Section 3. Each algorithm was trained for a fixed number of episodes sufficient for convergence, and the reported curves are smoothed using a moving average to aid visualization. Key hyperparameters (learning rates, network sizes, etc.) are provided in Appendix Table 5. For each algorithm we logged the average reward per episode and a system stability metric, as well as per–agent outcomes. Figure 10 provides an overview of the training performance curves for the three methods.

### 5.1 Benchmarking against other MARL methods

To place our approach in a broader MARL context, we additionally evaluated several state-of-the-art algorithms beyond MADDPG. Specifically, we trained MAPPO, MATD3, and MAAC on the same demographic crisis environment, as well as their evolutionary variants (MAPPO–EVO, MATD3–EVO, and MAAC–EVO). A moving-average comparison of the average reward and stability metrics for all nine methods is presented in Appendix B.2, along with detailed diagnostic plots for each evolutionary variant. These supplementary results confirm that the proposed evolutionary booster improves sample efficiency and final performance not only for MADDPG but also for other widely used MARL algorithms.

### 5.2 Performance comparison

Figure 10 shows that the baseline MADDPG converges slowly and attains a lower final reward (around 240), whereas the evolutionary variants achieve substantially better outcomes. MADDPG–EVO experiences sharp jumps in reward when evolutionary boosters are triggered and converges near 250. The adaptive MADDPG–EVO–DGM triggers its first booster earlier and ultimately reaches the highest reward (252.57), a modest 3.97% above the baseline but with a markedly faster ascent. For example, the EVO variant reaches an average reward of 150 after roughly 30 episodes, whereas the baseline requires around 800 episodes to do so, representing an improvement in sample efficiency of about 29%. Overall, evolution increases early–training reward by roughly 3.5 × and significantly accelerates convergence; the DGM meta–learning speeds up initial progress but yields only a small additional asymptotic gain.

All methods eventually achieve similar levels of population stability (inverse coefficient of variation around 0.71–0.72), but the evolutionary approaches reach this regime much more quickly and with less variability. The baseline begins with highly volatile dynamics and gradually stabilizes, whereas MADDPG–EVO and MADDPG–EVO–DGM rapidly reduce variability once the first evolutionary boost occurs. These methods also exhibit much lower run–to–run variance in final performance (coefficient of variation ≈1.4 × 10^−2^ vs. 3.8 × 10^−2^ for the baseline), indicating more reliable convergence.

## 6 Discussion

Our results demonstrate that a MARL approach augmented with evolutionary search can effectively learn complex demographic management policies under crisis conditions. The emergent behaviors suggest implicit coordination: although agents do not communicate explicitly, the shared reward structure incentivises them to avoid purely selfish actions that harm the collective. Evolutionary boosters accelerate the discovery of coordinated strategies by breaking symmetry and encouraging specialization among agents. For policy–makers, such learned strategies provide insights into how regions might balance growth and stability during crises—for example, which regions should prioritize retaining population versus which should accept losses for the greater good. The hybrid learning process essentially uncovered a form of *dynamic burden–sharing* between regions that a centralized planner or static model might not anticipate.

Several limitations remain. First, computational constraints limited active training to eight agents; scaling to all 89 regions would require further engineering (e.g., parameter sharing across similar regions, or factorized critics to reduce complexity). However, our approach is designed with scalability in mind (shared networks, etc.), and we anticipate that with more computing power or distributed training techniques, it can handle larger agent populations. Second, our crisis scenarios are stylised and the four-dimensional action space is still highly aggregated; real-world applications would require richer models with more detailed levers for fertility, mortality, migration, housing, healthcare capacity, labor-market measures, and fiscal constraints. For instance, actual crises could have complex feedback loops not captured by simple percentage modifiers. Third, the current implementation uses annual time steps because the regional demographic and socio-economic inputs are annual and because the main computational burden is MARL training. A quarterly or half-yearly version is technically possible by rescaling the transition step to Δ*t* = 0.25 or Δ*t* = 0.5, converting annual rates to period rates, and recalibrating crisis durations and lag parameters; however, we do not report subannual experiments here because they would require a new full training and validation cycle. Fourth, the booster hyperparameters (interval, noise scale, population size) were chosen empirically for our environment; an adaptive scheme or hyperparameter optimisation (e.g., Bayesian tuning or AutoML) could further improve efficiency. Our DGM–inspired adaptive interval is a step in this direction, but more sophisticated self–tuning mechanisms are possible (e.g., the agent could learn when to mutate based on an internal meta–reward for improvement). Fifth, interpretability remains a challenge: understanding *why* a particular policy emerges requires deeper analysis of the learned value functions and state–action trajectories. Tools from explainable RL or causal inference could be applied to translate policies into human-understandable rules. Finally, computational cost is non-trivial–MADDPG-EVO-DGM requires substantially more computation per training step than the baseline MADDPG, due to evaluating populations of mutants. This is manageable for eight agents, but scaling up will necessitate optimisations (e.g., parallel rollouts on GPU clusters, or intermittent usage of the evolutionary module in a hybrid mode as needed).

Despite these limitations, MADDPG–EVO–DGM lays a foundation for AI–assisted demographic policy design. The combination of multi–agent reinforcement learning and evolutionary optimisation enables policies that are robust to complex crises and heterogeneous regional conditions. To our knowledge this work is the first to successfully apply MARL at this scale in computational demography, and the first to integrate Darwin–Gödel Machine concepts into MARL. It suggests that hybrid learning methods can provide valuable insights for crisis–resilient demographic policy, where purely statistical or equilibrium models fall short.

Future work could extend the framework in several directions. One promising avenue is meta–learning extensions that allow agents to rapidly adapt to novel crises (e.g., a “meta–policy” that could adjust behavior if a never–before–seen shock occurs). Another is incorporating more hierarchical decision–making structures—for example, modeling not just regional governments but also a federal government agent that allocates resources or coordinates regions (this could reflect the real hierarchical governance in many countries). This might improve global outcomes and reflect the reality of multi–level policy responses. Additionally, scaling to a global setting (with multiple countries or hundreds of regions) and including international migration flows would broaden the applicability of the model. Lastly, integrating scenario-generation modules to produce plausible crisis scenarios (or even policy suggestions) could enrich the environment and could allow the agents to be tested against a wider range of stress-test narratives. In the long run, we envision that developing this approach further might lead toward a full–fledged Darwin–Gödel Machine for socio-demographic systems: a self-evolving decision–support system that can adapt to any challenges, even unforeseen “black swan” events, with the ultimate goal of preserving human lives and societal well-being through intelligent policy–making.

## 7 Conclusion

We introduced MADDPG–EVO–DGM, a hybrid multi–agent reinforcement learning algorithm enhanced with evolutionary optimisation and meta–learning, for modeling demographic and migration processes under multiple concurrent crises. In a calibrated simulation of Russia's regional population dynamics (eight regions, 2000–2024) subject to pandemics, conflicts, and economic downturns, our agents learned to coordinate policies that substantially improved population outcomes and system stability. Evolutionary boosters were crucial in escaping local optima, yielding faster convergence and higher rewards—early performance jumps of over +150% and a final average reward approximately 4% above the baseline, with significantly reduced variability. To our knowledge this is the first application of MARL in this domain and at this scale. Our work suggests that hybrid learning frameworks combining gradient–based and evolutionary methods can overcome challenges of non–stationarity and delayed rewards in complex social systems, providing a novel tool for crisis–resilient demographic policy design. We hope this approach stimulates further exploration at the intersection of AI and demography, ultimately guiding the development of adaptive governance systems that can safeguard populations in an era of uncertainty.

## Data Availability

The original contributions presented in the study are included in the article/supplementary material, further inquiries can be directed to the corresponding author.
