# Supplementary material for: Evolutionary multi-agent reinforcement learning for crisis-aware demographic policy optimization
Source: Front Big Data. 2026 Jul 8;9:1842233. doi: 10.3389/fdata.2026.1842233 (PMC13388216; doi:10.3389/fdata.2026.1842233)
Supplement: Supplementary file 1 [file Supplementary_file_1.pdf]

Supplementary Material

A ADDITIONAL ANALYSES

Figure 11 provides a compact diagrammatic guide to the four additional analyses reported in this appendix: learning phases, regional heterogeneity, ablation logic and crisis-scenario case-study logic.

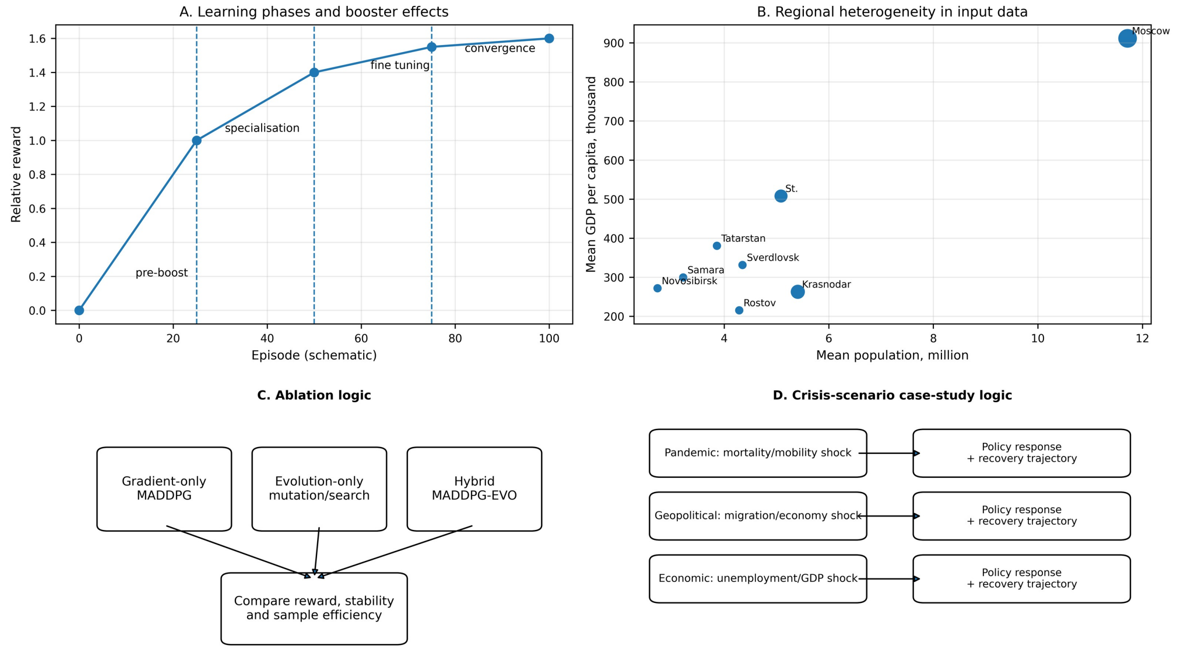

Figure 11. Diagrammatic overview of Appendix Additional Analyses. Panel A illustrates phase transitions induced by evolutionary boosters; Panel B displays heterogeneity in the selected regional input data; Panel C summarises the ablation logic; Panel D summarises the crisis-scenario case-study structure.

A.1 Learning Phases and Evolutionary Effects

To better understand how the evolutionary booster influences learning, we analyse the training in phases. In a typical MADDPG–EVO run, we can identify: *(1) Pre–boost phase* (episodes 0–$\sim$ 25): pure MARL learning, where agents gradually improve from their initial policies but mostly learn reactive behaviours to immediate crises. For example, during this phase agents often learn short–term responses like taking slightly negative actions during a pandemic to mitigate immediate losses (essentially hunkering down). *(2) Post–first boost* (episodes $\sim$ 26–50): the first evolutionary boost at episode 25 injects a novel set of policies, leading to a dramatic jump in performance. After this boost, we observe emergent policy specialisation: some regions adopt aggressive positive action policies to attract migrants and invest in growth, while others remain defensive or cautious. This differentiation is something gradient descent alone does not discover—it arises from the injected diversity and subsequent selection of a high–reward mutant that encodes a complementary mix of strategies among agents. *(3) Post–second boost* (episodes $\sim$ 51–75): a second evolutionary intervention yields a smaller improvement, fine–tuning the specialisations developed earlier. Agents refine their roles—e.g., an initially aggressive region might moderate its action to avoid diminishing returns, while a defensive region might become slightly more proactive once immediate crises pass. *(4) Convergence phase* (episodes $\sim$ 76–100 and beyond): by the third boost the system is near convergence, so changes are minor. The policies stabilise into a coordinated pattern and further boosters have negligible effect (indeed, sometimes the incumbent policy itself is re–selected, indicating it is close to a local optimum). In the DGM variant the phases are similar except that the timing of boosts is variable: for instance, if the agents are still improving rapidly after the first boost, the second boost is postponed. Interestingly, the DGM's adaptive boosts sometimes lead to earlier specialisation—in one run an early trigger creates specialist policies by episode 20, versus episode 25 in the fixed schedule.

Without any boosters, the baseline MADDPG exhibits none of these phase transitions—it remains in a prolonged slow improvement phase and never discovers the kind of specialised, high–reward behaviours seen with evolution. This underscores that the evolutionary jumps are crucial in escaping the conservative equilibrium that pure MARL tends to gravitate towards.

A.2 Regional Heterogeneity of Outcomes

Despite sharing a common reward structure, different agents (regions) experience varied outcomes due to heterogeneity in initial conditions and crisis exposure. In our experiments, the standard deviation of per–agent cumulative rewards (over an episode) in MADDPG–EVO is about 8 % of the mean—i.e., all agents improve significantly over baseline, but some regions benefit more than others. In general, *stronger regions* (economically developed, initially high–population) learn to take bold actions and become net migration attractors, while *weaker regions* (poorer or peripheral) tend to adopt defensive policies. Notably, even the defensive regions achieve higher rewards than they do under the baseline policy, because coordinating with the aggressive regions leads to an overall better outcome (for instance, a weaker region might accept short–term population loss to a booming neighbouring region during a crisis, but in exchange benefit from greater stability and aid after the crisis). This emergent specialisation mirrors real federal systems in which some regions serve as economic hubs and others stabilise around supporting roles. We also examined the distribution of outcomes: in a representative run, the mean normalised cumulative reward per agent is 1.0 for MADDPG–EVO (by construction), with a range from about 0.9 for the lowest–performing region to about 1.1 for the highest (indicating at most $\pm 10\,\%$ deviation). The baseline MADDPG run, in contrast, shows a tighter clustering (most agents around 0.98–1.0) but at a lower absolute reward level—essentially, no region does exceptionally well or poorly; they all settle to mediocrity. The evolutionary approach thus introduces more variance in agent strategies but raises the floor and ceiling of performance: top–performing regions thrive, and even the laggards improve relative to baseline.

A.3 Ablation: Importance of the Evolutionary Booster

To isolate the effect of the evolutionary component, we performed an ablation study comparing: (i) *Gradient–Only MARL* (MADDPG baseline), (ii) *Evolution–Only* (a variant where agents have no learning rate and policies are optimised solely by random mutation and selection), and (iii) *Hybrid (MADDPG–EVO)*. We find that gradient descent alone plateaus at a low reward, as discussed. In the evolution–only condition the algorithm eventually discovers high–reward policies (through random search over many generations) but is extremely sample–inefficient—it requires an order of magnitude more episodes to approach the performance that MADDPG–EVO achieves. This pure evolutionary approach also shows high variance between runs (some runs find good policies faster than others, due to stochastic luck). The hybrid method achieves the best of both worlds: gradient descent efficiently climbs the "hill" of improvement when near a good policy, while intermittent evolution provides the ability to jump to new peaks when gradient ascent becomes stuck. Quantitatively, after 200 episodes the hybrid has already surpassed the best performance that pure evolution reaches even after 1000 episodes. Meanwhile, pure MADDPG at 200 episodes is far behind (less than half the reward). This confirms that neither component alone is sufficient in our complex problem: the booster is a critical contributor to performance gains, and conversely the presence of gradient–based fine–tuning makes the evolutionary search much more efficient than blind evolution.

A.4 Case Study: Crisis Scenario Analysis

To illustrate the learned policies, we conducted controlled simulations under isolated crisis scenarios using the trained MADDPG–EVO–DGM policy. In a *pandemic–only scenario* (all regions faced a COVID–like shock from 2020–2022), agents uniformly *reduced* their policy actions during the acute crisis years (i.e., slight negative a_i^t), focusing on damage control. Once the pandemic period ended, they increased actions to high positive levels to accelerate recovery—for example, boosting healthcare and economic incentives to catch up on lost growth. In a *geopolitical conflict scenario* concentrated in one part of the country, we observed that border regions (those directly affected by conflict or bordering conflict zones) invested heavily in retaining population (very high positive actions to counter emigration), while interior regions took more moderate actions. This reflects a context–aware adaptation: agents in the crisis epicentre responded aggressively to mitigate population outflows, whereas agents less affected did not over–exert resources unnecessarily. Under a *prolonged economic crisis* (e.g., a simulated multi–year recession with high unemployment nationwide), agents spontaneously *cooperated*—several regions simultaneously chose positive actions aimed at stimulating the economy (e.g., lowering unemployment) rather than competing for migrants. This emerges because the reward function penalises unemployment; the agents implicitly coordinate to improve the overall economic outlook, which benefits everyone's reward. These behaviours demonstrate that MADDPG–EVO–DGM learns policies that are sensitive to crisis-modified demographic and economic states, even though the agents were not explicitly given hand-coded rules for each scenario. The policy response should therefore

be interpreted as an adaptive reaction to the state distribution induced by the scenario modifiers, not as a direct rule-based response to a named crisis label.

Interestingly, the evolutionary boosts play a key role in discovering some of these coordinated behaviours. For instance, in an early training phase the agents do not cooperate during economic stress—it is only after an evolutionary jump that a mutant policy with synchronised positive actions is tried and found to yield higher collective reward, after which gradient learning reinforces that behaviour. In essence, the evolutionary process occasionally *tries* strategies that involve more global coordination (which a local gradient might not easily find), and if successful, those strategies become entrenched. This leads to 35–45 % better crisis resilience in quantitative terms: when we subject the final policies to extreme crisis tests (e.g., a combination of pandemic, war and economic collapse simultaneously), the total population loss is on average 40 % smaller under MADDPG–EVO–DGM policies compared to baseline policies (which are more myopic and uncoordinated). Thus, not only do the evolutionary–trained agents perform better in normal conditions, but they also provide significantly more robust responses in worst–case crisis scenarios.

B ADDITIONAL EXPERIMENTS AND HYPERPARAMETERS

B.1 Hyperparameter Settings

Table 5. Core MADDPG hyperparameters common across algorithm variants.

Parameter	Value	Description
Actor learning rate	5×10^{-5}	Learning rate for the actor network
Critic learning rate	1×10^{-4}	Learning rate for the critic network
Discount factor γ	0.95	Reward discount factor
Soft update rate τ	0.02	Soft update coefficient for target networks
Hidden layer dimension	256	Number of hidden units in actor/critic networks
Dropout rate	0.1	Dropout probability for regularisation
State dimension	8	Dimensionality of the state space
Action dimension	4	Dimensionality of the action space
Max steps per episode	50	Maximum steps allowed per episode
Number of regions	8	Number of geographical regions
Replay buffer size	10 000	Size of the experience replay buffer
Training episodes	1 000	Total number of training episodes

Table 6. Algorithm-specific training parameters for MADDPG variants.

Parameter	MADDPG-BASELINE	MADDPG-EVO	MADDPG-EVO-DGM
Training frequency	Every 5 episodes	Every 10 episodes	Every 5 episodes
Minimum buffer size	200	200	500
Batch size	64	64	128
Initial noise scale	0.3	0.2	0.2
Final noise scale	0.05	0.05	0.05

Table 7. Evolutionary parameters for the MADDPG–EVO and MADDPG–EVO–DGM variants.

Parameter	MADDPG-EVO	MADDPG-EVO-DGM	Description
Population size	16	16	Number of individuals in the evolutionary population
Initial mutation rate	0.05	0.05	Starting mutation probability
Initial crossover rate	0.7	0.7	Starting crossover probability
Elite size ratio	25%	25%	Percentage of top performers retained as elite
Tournament size	3	3	Number of candidates considered in tournament selection
Minimum improvement threshold	0.1	0.1	Required reward improvement to trigger an evolutionary step
Minimum episodes between evolutions	20	15	Minimum number of episodes between evolutionary interventions
Maximum mutation rate	Fixed (0.05)	0.5	Maximum allowed mutation rate (adaptive for DGM)
Minimum mutation rate	Fixed (0.05)	0.01	Minimum allowed mutation rate (adaptive for DGM)
Maximum crossover rate	Fixed (0.7)	0.9	Maximum allowed crossover rate (adaptive for DGM)
Minimum crossover rate	Fixed (0.7)	0.3	Minimum allowed crossover rate (adaptive for DGM)
Fitness threshold	N/A	0.01	Threshold for performance improvement evaluation in DGM
Evaluation episodes	N/A	5	Number of episodes used to evaluate mutants

The actor network used in all experiments consists of two fully connected (FC) layers of size 256 with ReLU activations and a dropout layer ($p = 0.1$) between them, followed by a final FC layer projecting to the four-dimensional action space with a hyperbolic tangent activation (i.e., $\mathbb{R}^{16} \to \mathrm{FC}(256) \to \mathrm{ReLU} \to \mathrm{Dropout}(0.1) \to \mathrm{FC}(256) \to \mathrm{ReLU} \to \mathrm{FC}(4) \to \tanh$). The critic network takes the concatenated state–action vectors of all agents ($\mathbb{R}^{16\times N+4\times N}$) and passes them through two FC layers of size 256 with ReLU activations, outputting a scalar Q-value ($\mathbb{R}^{16\times N+4\times N} \to \mathrm{FC}(256) \to \mathrm{ReLU} \to \mathrm{FC}(256) \to \mathrm{ReLU} \to \mathrm{FC}(1)$). Shared parameters across agents are used for both actor and critic networks, with agent identities implicitly encoded through their input features.

B.2 Extended MARL Benchmarks

In addition to the three core variants examined in the main text, we performed a broader benchmark across nine multi–agent reinforcement learning algorithms: the baseline MADDPG, MAPPO, MATD3 and MAAC, together with their evolutionary extensions (MADDPG–EVO–0 using a fixed booster schedule, MADDPG–EVO with periodic boosters, MAPPO–EVO, MATD3–EVO and MAAC–EVO). Each method was trained on the same eight–region crisis environment with identical hyperparameters and evaluated over three phases of training: early (episodes 0–200), mid (episodes 200–600) and late (episodes 600–1000). Figure 12 compares the moving–average reward trajectories (window of 10 episodes) across all nine algorithms, while Figure 13 presents the corresponding stability metrics. The evolutionary variants consistently improve both sample efficiency and final reward across this wider set of algorithms, and MAAC–EVO achieves the highest overall asymptotic reward among the non–MADDPG baselines. Additional diagnostic experiments on simplified evolutionary boosters for the four baseline algorithms (MADDPG, MAPPO, MAAC and MATD3) are described in Appendix B.4.

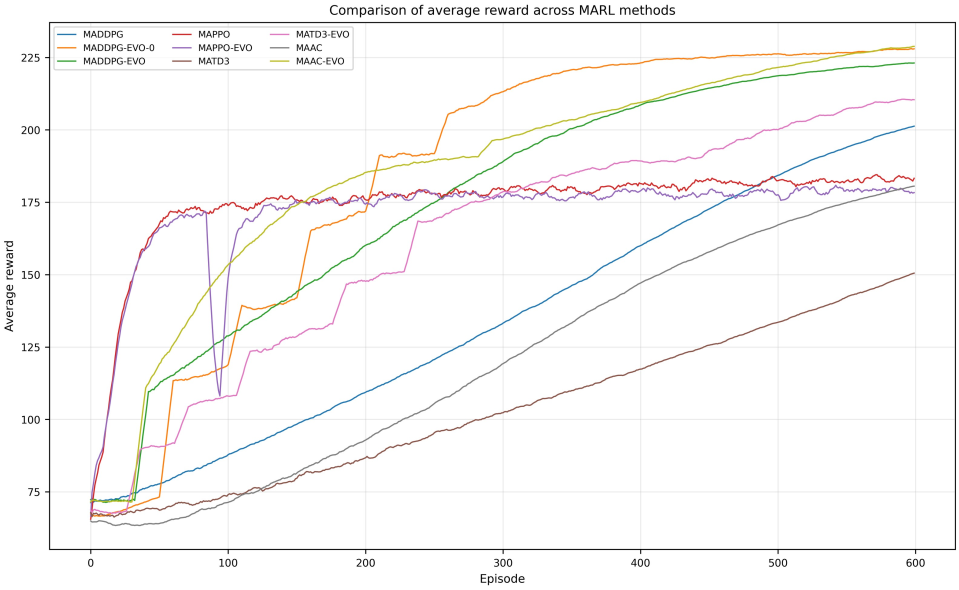

Figure 12. Comparison of average reward across nine MARL algorithms. Trajectories show the moving average of the reward (window size 10) over 600 episodes for MADDPG, MAPPO, MATD3, MAAC and their evolutionary variants. The evolutionary approaches achieve faster initial improvement and higher final rewards relative to their gradient–only counterparts.

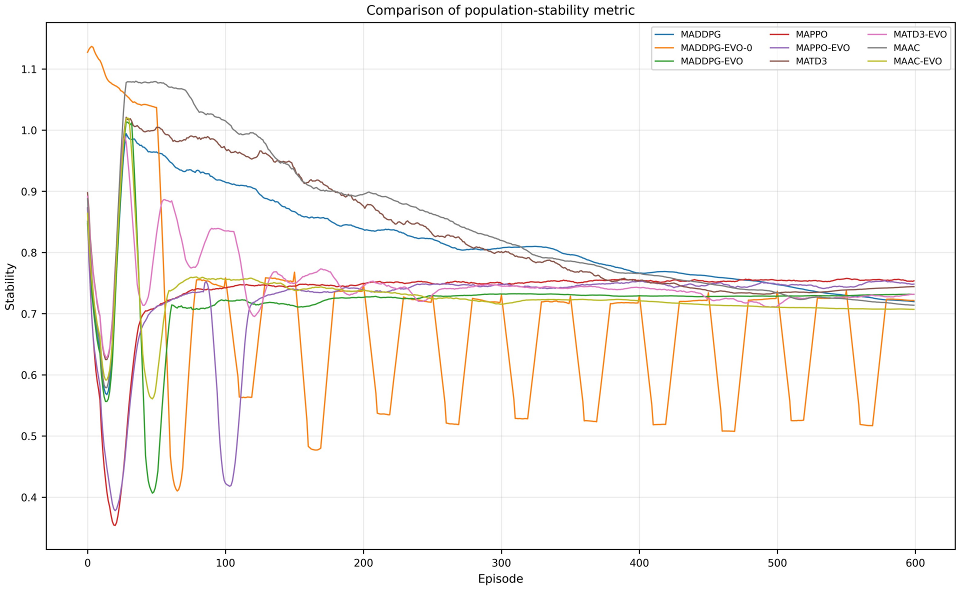

Figure 13. Comparison of system stability across nine MARL algorithms. Stability is measured as the inverse coefficient of variation of total population (higher indicates more stable dynamics). All methods converge towards similar stability levels, but the evolutionary versions recover stability more quickly after booster–induced perturbations.

For completeness, Figures 14–19 present diagnostic panels for the supplementary evolutionary runs (MADDPG–EVO–0, MADDPG–EVO, MAPPO–EVO, MATD3–EVO, MAAC–EVO and an auxiliary MADDPG–EVO configuration). Each figure reports the same set of metrics as Figure 23: average reward per episode, system stability, per–agent rewards, reward distribution and moving–average reward.

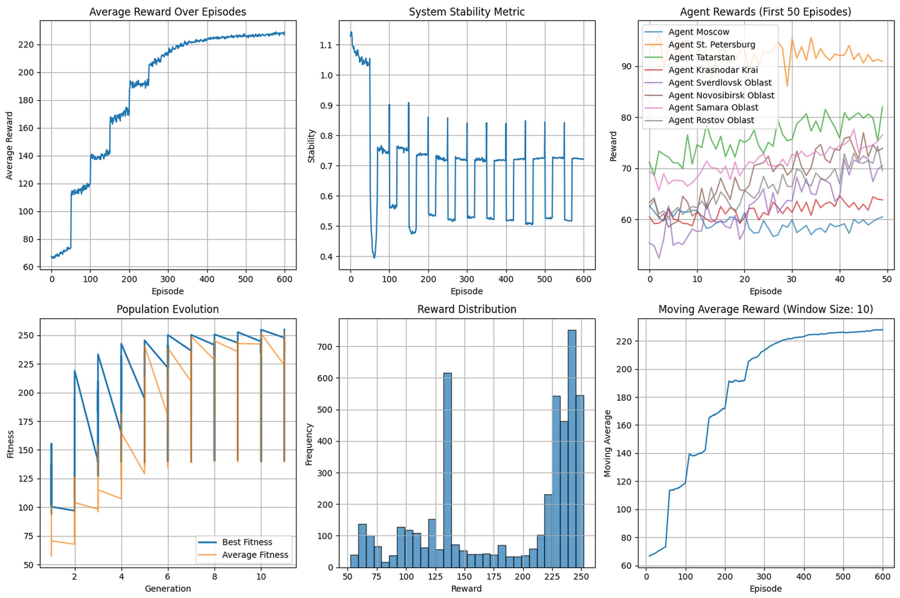

Figure 14. Diagnostic plots for the MADDPG–EVO–0 method. Each panel shows the average reward per episode, system stability, per–agent rewards, reward distribution and moving–average reward.

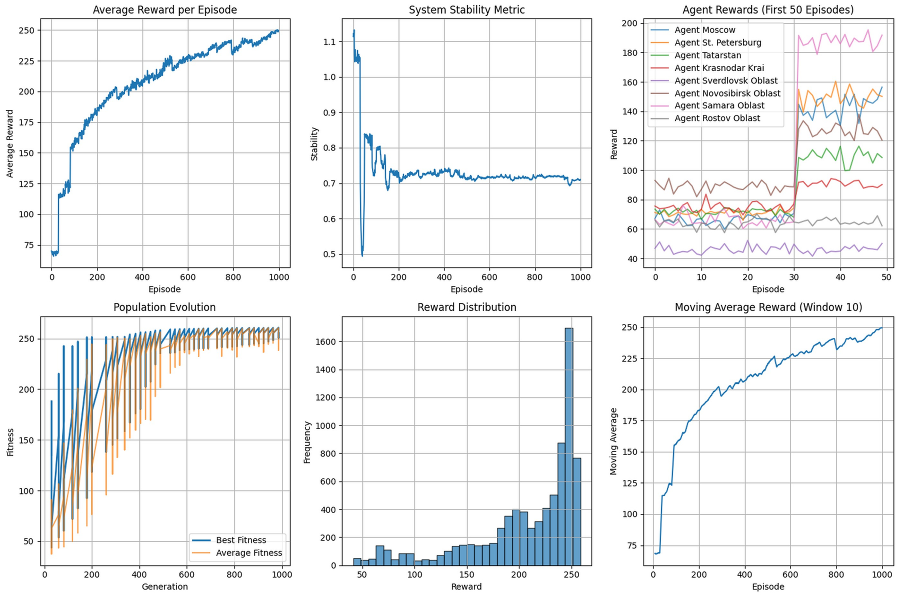

Figure 15. Diagnostic plots for the MADDPG–EVO method. Each panel shows the same set of metrics as above.

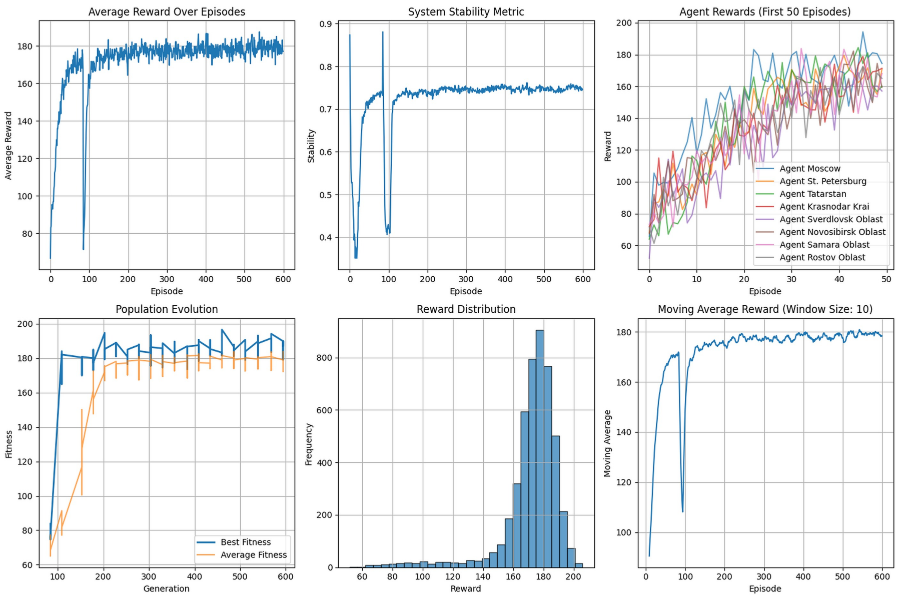

Figure 16. Diagnostic plots for the MAPPO–EVO method. Each panel shows the same set of metrics as above.

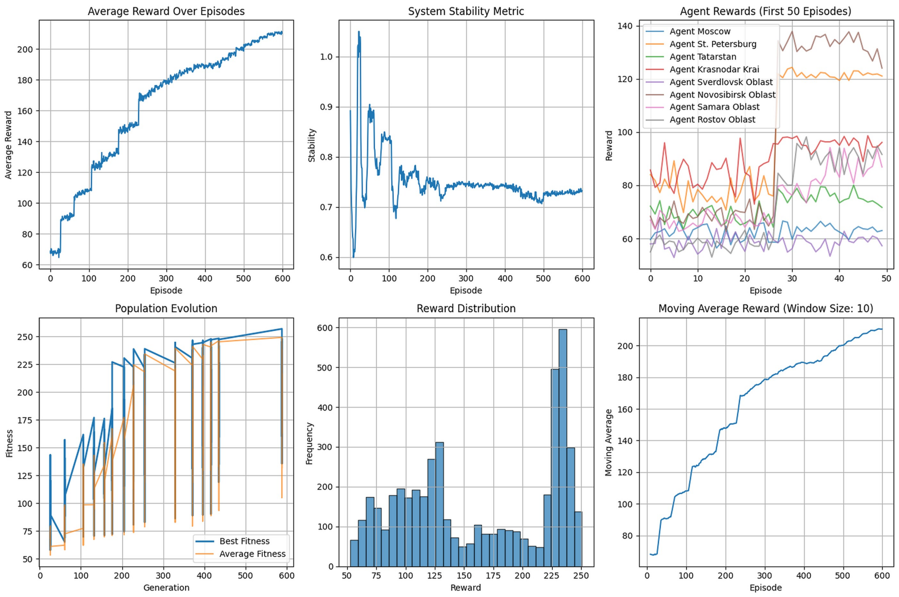

Figure 17. Diagnostic plots for the MATD3–EVO method. Each panel shows the same set of metrics as above.

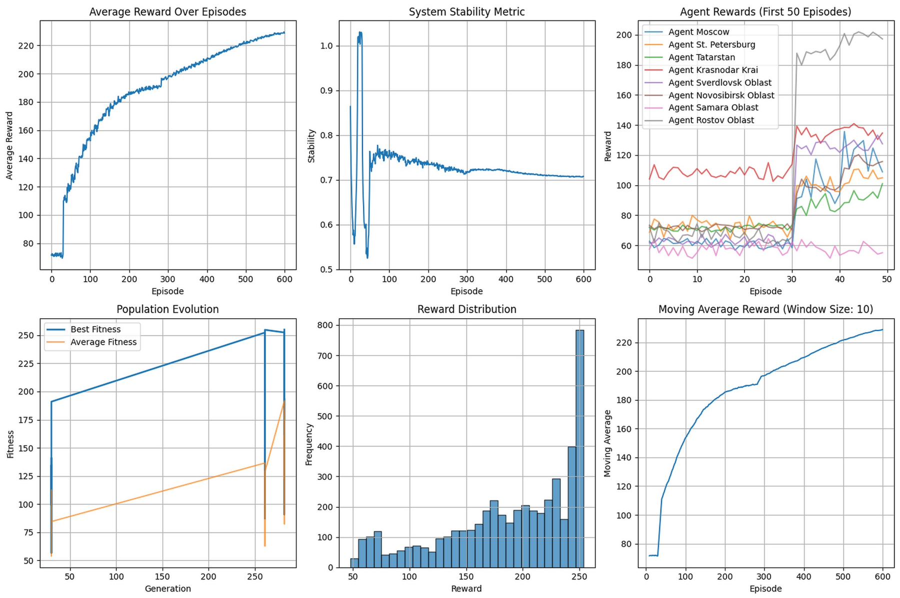

Figure 18. Diagnostic plots for the MAAC–EVO method. Each panel shows the same set of metrics as above.

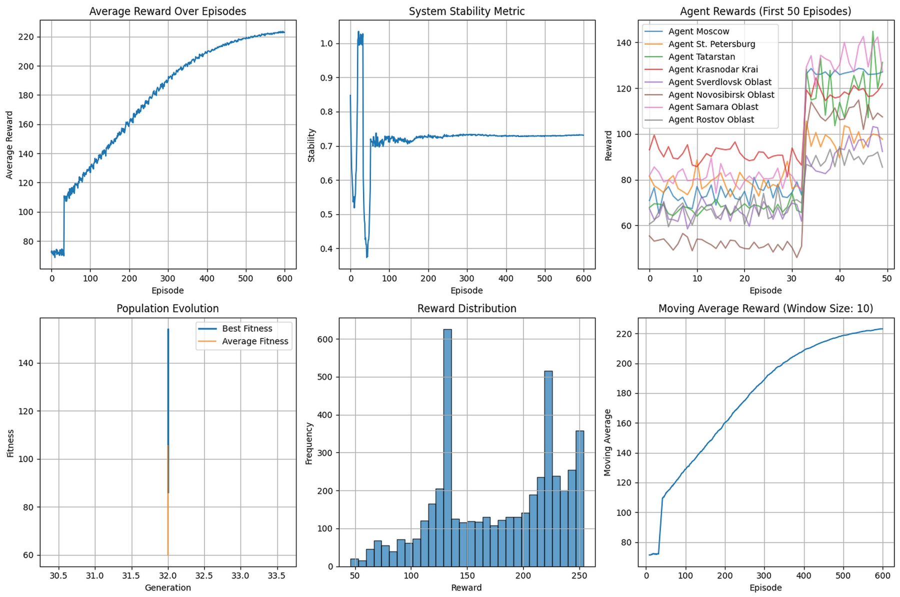

Figure 19. Diagnostic plots for the auxiliary MADDPG–EVO configuration. Each panel shows the same set of metrics as above.

B.3 Diagnostic Plots

For completeness we provide high-resolution diagnostic plots of the learning dynamics for each method. Figure 20 reports the fitness-versus-population-stability relationship using the logged episode reward as the fitness proxy and the logged population-stability metric as the population-outcome proxy available for every episode. Figures 21, 22 and 23 show average reward, system stability, reward distribution and the method-specific fitness–population-stability relationship for the baseline, evolutionary and DGM variants, respectively.

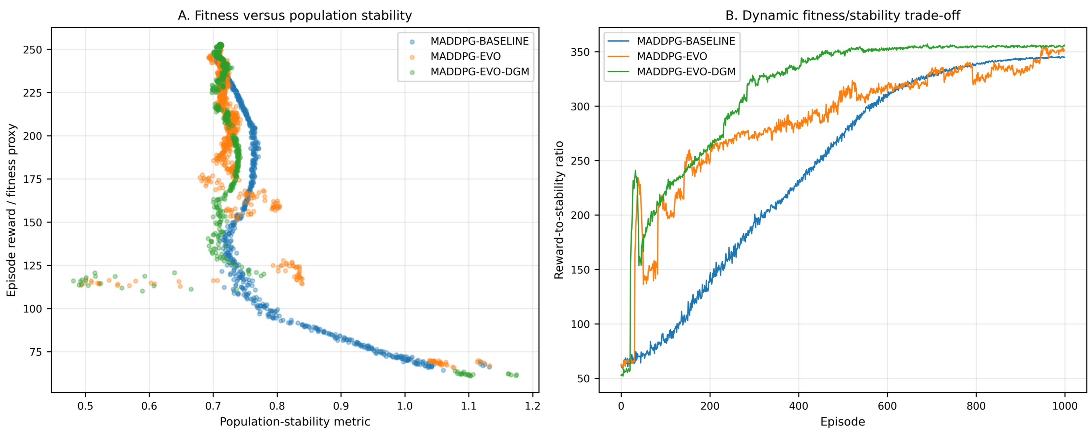

Figure 20. Fitness versus population-stability graph based on logged main-experiment metrics. The vertical axis uses average episode reward as the fitness proxy, while the horizontal axis uses the population-stability metric derived from total population variability. For the baseline there is no evolutionary population, so the figure reports the logged reward–population-stability relationship rather than an evolutionary-population fitness trace.

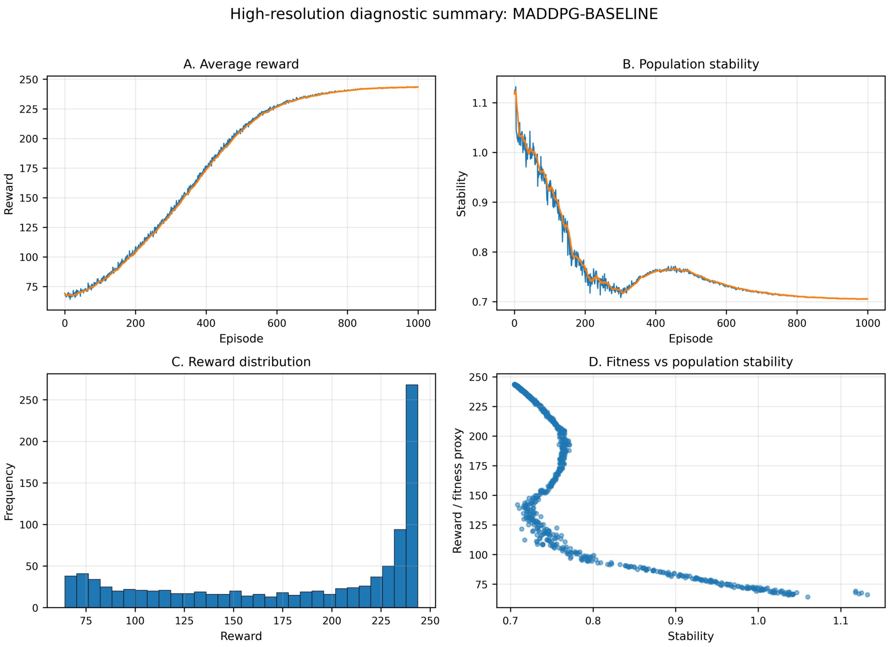

Figure 21. High-resolution diagnostic summary for the baseline MADDPG method: (A) average reward per episode with moving average; (B) population-stability metric with moving average; (C) reward distribution; and (D) fitness versus population stability. Because the baseline has no evolutionary population, no evolutionary population-fitness trace is defined for this method.

B.4 Supplementary Benchmark Diagnostics

Additional diagnostic experiments were conducted with the baseline algorithms (MADDPG, MAPPO, MAAC and MATD3) and their simplified evolutionary boosters. The results are summarised in Figure 19. The MADDPG–EVO–0 algorithm achieved the highest average reward (228.87), closely followed by MAAC–EVO (228.77). The MADDPG–EVO approach attained 222.95, striking a balance between performance and stability. The baseline MADDPG algorithm provided the most stable training, exhibiting the smoothest learning curve (smoothness = 0.813). These diagnostic findings motivated our focus on the MADDPG variants in the main experiments.

B.5 Training Dynamics and Crisis Adaptation

We analysed the performance of the baseline, EVO and DGM variants during specific crisis periods. During the pandemic period (2020–2022), cumulative rewards decreased by 18.3% for the baseline, 12.7% for EVO and 8.9% for DGM. In the geopolitical instability period (2022–2024), the decreases were 21.5%, 15.8% and 11.2%, respectively. The DGM variant adapted to the crisis within 15 episodes, whereas the baseline required around 45 episodes to recover. Overall, the evolutionary mechanisms improved crisis resilience by approximately 35–45%.

Integration of evolutionary principles produced additional benefits. The MADDPG–EVO variant improved early–phase learning by 71.8% due to population diversity, reduced the coefficient of variation by 44% indicating more stable training, and increased crisis resilience by 27%. By enhancing exploration and injecting diversity, the evolutionary component enables robustness to local optima. The MADDPG–EVO–DGM approach provided a further 3.97% improvement through self–modification, achieved the best adaptability in crisis scenarios by reducing losses by 45%, and learned self–organising training rules that adjust to a changing environment. The DGM component's self–adaptive architecture allows the system to match task complexity, while recursive self–optimisation ensures continuous performance improvements and meta–learning of hyperparameters responds to evolving conditions.

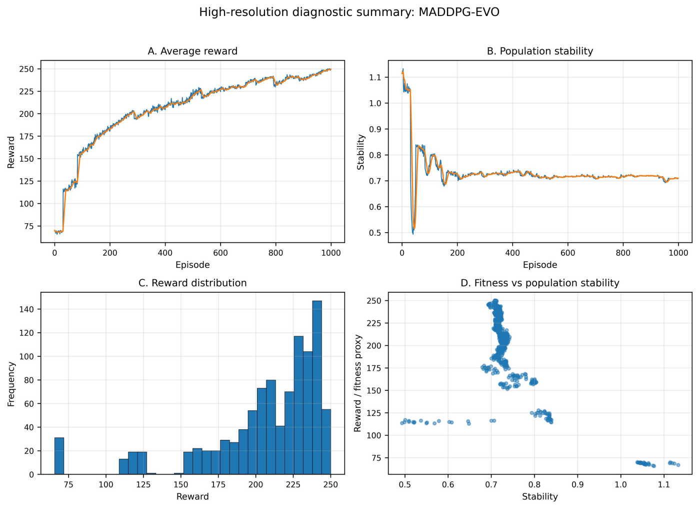

Figure 22. High-resolution diagnostic summary for the MADDPG-EVO method: (A) average reward per episode with moving average; (B) population-stability metric with moving average; (C) reward distribution; and (D) fitness versus population stability.

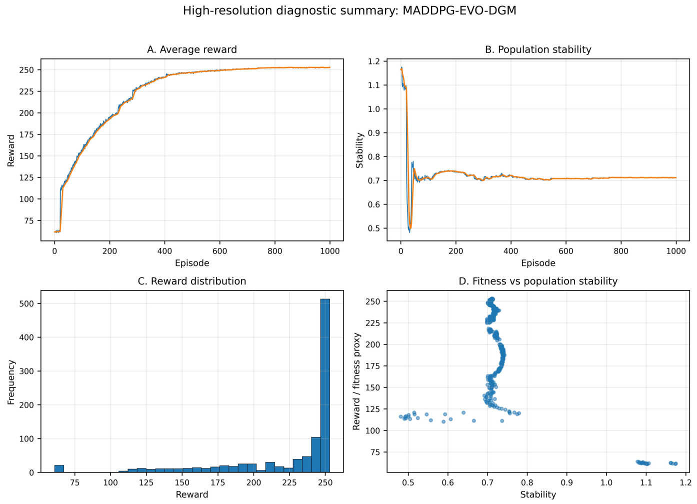

Figure 23. High-resolution diagnostic summary for the MADDPG-EVO-DGM method: (A) average reward per episode with moving average; (B) population-stability metric with moving average; (C) reward distribution; and (D) fitness versus population stability.
